# Chronic Periodontitis Case Definitions and Confounders in Periodontal Research: A Systematic Assessment

**DOI:** 10.1155/2018/4578782

**Published:** 2018-11-28

**Authors:** Zuhair S. Natto, Randa H. Abu Ahmad, Lina T. Alsharif, Hanan F. Alrowithi, Duaa A. Alsini, Hetaf A. Salih, Nabil F. Bissada

**Affiliations:** ^1^Department of Dental Public Health, School of Dentistry, King Abdulaziz University, Jeddah, Saudi Arabia; ^2^Department of Periodontology, School of Dental Medicine, Tufts University, Boston, MA, USA; ^3^Department of Oral Health Policy and Epidemiology, School of Dental Medicine, Harvard University, Boston, MA, USA; ^4^Department of Periodontics, School of Dental Medicine, Case Western Reserve University, Cleveland, OH, USA

## Abstract

Case definitions and criteria of periodontal diseases are not yet consistent worldwide. This can affect the accuracy of any comparison made between two studies. This study determines which are the most common chronic periodontitis case definitions as well as confounding variables that have been reported worldwide in periodontal literature. A systematic assessment on periodontal disease classification and confounders was conducted using all publications in MEDLINE, EMBASE, SCOPUS, and Google Scholar between 1965 and October 2017. Screening of eligible studies and data extraction were conducted in duplicate and independently by two reviewers. The search protocol produced 4,218 articles. Out of these, 492 potentially relevant articles were selected for review. Only 351 studies fulfilled the selection criteria. Combination of probing depth and clinical attachment loss was the most common chronic periodontitis case definitions used (121, studies, 34.5%). CPI/CPITN was the most common classification used. Age was the most common confounder studied in periodontal research (303 studies, 86.3%), followed by gender (268 studies, 76.4%) and race (138 studies, 39.3%). Albumin and creatinine were the least common variables studied (1 or 2 studies each). Different case definitions affect the prevalence and treatment consequences of periodontitis. We need to standardize periodontitis case definitions worldwide to avoid difficulties in case diagnosis and prognosis. Further studies need to be done to assess the association between periodontitis and several potential confounders.

## 1. Introduction

Periodontal disease and dental caries are the most common diseases in the oral cavity [1]. Chronic periodontitis is one of the periodontal diseases. It is a long-lasting inflammatory disease affecting the soft and hard tissues around the teeth [2] and it is common worldwide [3]. This disease is related to common and preventable biological risk factors (e.g., high blood pressure, high blood cholesterol, diabetes, genetic factors, and obesity) and behavioral risk factors (e.g., an unhealthy diet, physical inactivity, and tobacco use) [4]. Based on published studies, the severity and prevalence of the disease vary significantly among populations [1]. It could be due to several reasons such as differences in data collection methods or case definitions of periodontal diseases.

Chronic periodontitis is ideally diagnosed at the beginning of the disease. However, case definitions and criteria that are used to diagnose this disease are not yet consistent worldwide [5–13]. This can affect the accuracy of any comparison made between studies. Kassab et al. applied different definitions of periodontitis to assess the disease in postpartum women. They found that case definitions had different impacts on the frequency of periodontitis, and they produced various odds ratios for the associations with risk factors for periodontitis [13]. Manau et al. found that a different case definition can change the statistical significance and effect size between periodontitis and prematurity or low birth weight [14].

Moreover, several studies have shown there are various risk factors associated with chronic periodontitis [1, 3, 15, 16]. However, recent factors have been investigated in published articles, and the number of associated confounders has increased dramatically since then. Furthermore, these reviews and articles have not described which variables are commonly investigated and which are not.

Thus, little is known about the common case definition of chronic periodontitis in epidemiological literature or the most common risk factors/predictors associated with such. So, the purpose of this systematic assessment was to assess the various definitions and factors of chronic periodontitis.

## 2. Materials and Methods

### 2.1. Search Strategies

We conducted a systematic literature review using all publications in MEDLINE, EMBASE, SCOPUS, and Google Scholar between 1965 and October 2017. The terms we used to identify epidemiological articles reporting on periodontist were the following: periodontal diseases (MeSH term and keyword), periodontal attachment loss (MeSH term and keyword), and periodontitis (MeSH term and keyword). These terms were combined with one of the following terms: prevalence (MeSH term), epidemiologic studies (MeSH term), epidemiology (MeSH term), epidemiologic research design (MeSH term), and risk (MeSH term). Hand-searched journals and bibliographies of the selected articles and reviews were checked for additional articles.

### 2.2. Eligibility Criteria

Included articles had to report the original article (i.e., letters to the editor and review studies were excluded); humans (i.e., animal and* in vitro* studies were excluded); and observational, population screening, or prevalence studies (i.e., case report, case series, and randomized clinical trial were excluded). The included chronic periodontitis definition and measurements were to be written in English only. We excluded articles describing gingivitis or other forms of periodontitis (e.g., aggressive and necrotizing ulcerative). Furthermore, we excluded any methodological and interventional studies as well as any studies for which the full text was not available/accessible through a license at our institutes.

### 2.3. Screening Process

Two reviewers (ZN) with either RA, LA, HA, DA, or HS independently screened and selected articles for eligibility based on title and abstract. Disagreements were resolved via discussion. After consensus, full-text articles were retrieved, and two reviewers (ZN) with either RA, LA, HA, DA, or HS screened the full-text articles and extracted data. In case of doubt, a new third reviewer in the same group (RA, LA, HA, DA, or HS) was involved.

### 2.4. Data Extraction

Items extracted from articles included study design (e.g., cohort and case-control), type of disease, case definition, method of diagnosis, and predictors including but not limited to the following: age, gender, family history, race, smoking, body mass index (BMI), height, weight, physical activity, diet, psychosocial status, socioeconomic status, alcohol, diabetes, cardiovascular disease, blood pressure, other diseases, cholesterol, blood glucose, and C-reactive protein.

To insure consistent data extraction, a standardized data extraction sheet was formed. It was tested and modified several times. All reviewers were trained on how to use the sheet and understand each component in it.

### 2.5. Descriptive Analysis

Results were summarized using descriptive statistics of frequencies and percentages. We did not perform a quantitative analysis, as this was beyond the scope of our review and meta-analysis for these definitions is inapplicable due to the heterogeneity and limited number of studies in several groups.

### 2.6. Patient Involvement

No patients were involved in any part of this study.

## 3. Results

**Figure 1:**
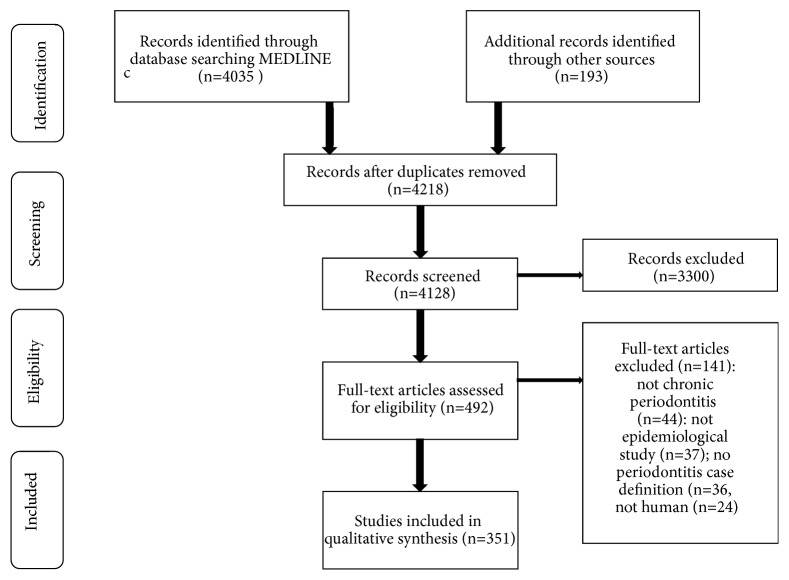
Flowchart of the systematic review.

The search strategy identified 4,218 unique articles, of which 3,726 were excluded based on title and abstract. In total, 492 full texts were screened, 451 of which met the eligibility criteria and were included in this review (Figure 1).

### 3.1. Case Definitions

**Figure 2:**
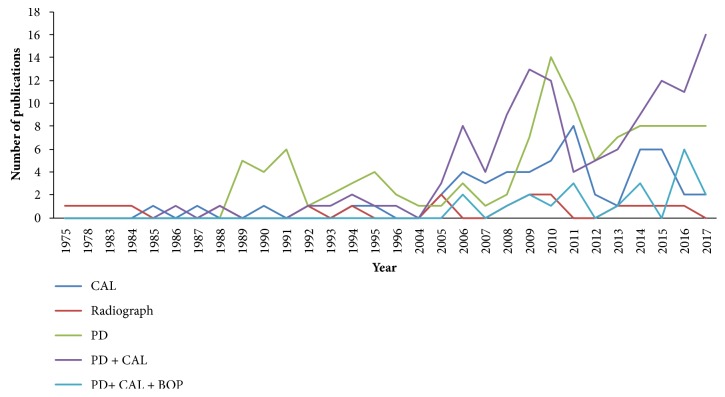
Numbers of articles based on the most common chronic periodontitis case definitions, ordered by publication year.

Overall, 121 (34.5%) articles used both probing depth (PD) and clinical attachment loss (CAL) combined, followed by PD only (110 studies, 31.3%), CAL only (54 studies, 15.4%), or radiograph only (19 studies, 5.4%) (Table 1). There are several methods that have been used rarely, such as the combination of CAL with furcation and PD with furcation (one study each). The combination of PD and CAL has been used more commonly in the recent 5 years, followed by PD (Figure 2).

Moreover, it is important to consider cases with CAL ≥ 3 periodontitis (21 studies, 6%), followed by CAL ≥ 1 (15 studies, 4.3%) and CAL ≥ 4 (11 studies, 3.1%) (Table 2). A minimum of two sites was the most common diagnostic criterion used. Bitewing was the most common method used in radiographic studies (11 studies, 3.1%), followed by a minimum of 2 mm (4 studies, 1.1). While PD ≥ 4 was considered enough to diagnose with periodontitis (93 studies, 26.5%), at least one site in this group was enough to diagnose with periodontitis (Table 2).

In the combination diagnostic criteria, PD ≥ 4 mm and CAL ≥ 3 mm were the most common with 34 studies (9.7%), followed by PD ≥ 5 mm, CAL ≥ 4 mm (26 studies, 7.4%), PD ≥ 4 mm with CAL ≥ 4 mm (14 studies, 4.0%), and PD ≥ 5 mm with CAL ≥ 6 mm (12 studies, 3.4%) (Table 2). At least 2 sites CAL and one site PD and at least 2 sites each were the most common in the category (36 studies, 16.1%, and 17 studies, 7.6%, respectively) (Table 2).

The results did not change with three criteria (e.g., PD, CAL, and BOP). PD ≥ 4 mm with CAL ≥ 3 mm and BOP was the most common (8 studies, 2.3%). At least four teeth with one or more site had 11 studies (4.9%) (Table 2).

### 3.2. Severity

There is no clear distinction between the three types of periodontitis (Table 3). However, it is more common to use Center for Disease Control/American Academy of Periodontology (CDC/AAP) working group classification, published by Eke et al. (42 studies), which divides cases as PD ≥ 5 and CAL ≥ 4 for moderate, PD ≥ 5 and CAL ≥ 6 for severe chronic periodontitis, and Armitage classification (15 studies), which divided CAL into 1–2 mm (mild), 3–4 mm (moderate), and CAL ≥ 5 mm (severe).

### 3.3. Predictors/Confounders

**Figure 3:**
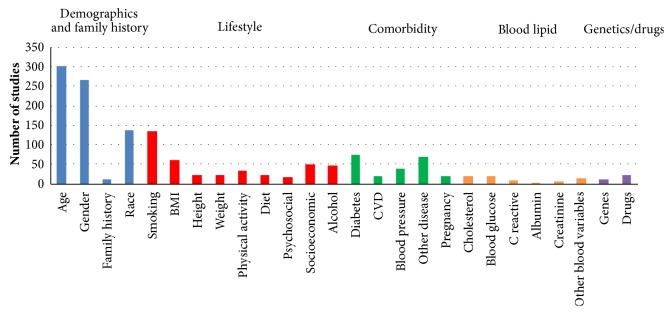
Predictors included in selected articles.

The median number of predictors included in articles was 3 (range 1-17). Age and gender were included in 303 articles (86.3%) and 268 articles (76.4%), respectively (Figure 3). Other prevalently selected predictors were race (138, 39.3%), smoking (135, 38.5%), diabetes (75, 21.4%), other diseases (71, 20.2%), and BMI (62, 17.7%). However, certain variables were included in few articles such as family history of periodontitis (11, 3.1%), genetics (11, 3.1%), C-reactive protein (9, 2.6%), creatinine (5, 1.4%), and albumin (2, 0.6%).

## 4. Discussion

The case definition for a disease is the key factor for any specialty. It is different from diagnosis because case definitions must be more quantitative, specific, accurately measurable, and relatively few in number [7]. This is an important issue in the periodontal research field [6–8, 10, 17–21]. We have shown in this article that there are different definitions for chronic periodontitis in the literature, which can affect estimates of prevalence, incidence, and treatment strategies. It is also clear that variation in threshold values—for CAL, PD, radiograph, or any combination at a given site—leads to different diagnosis of chronic periodontitis at that site. In addition, the number of involved sites [7, 12, 22]. Selection of threshold values is very critical and has been stated in several articles [7, 12, 22]. Any changes in these values or number of sites can lead to major changes in the prevalence scores, which may overestimate or underestimate the actual disease status.

Type and number of methods used is another factor which can affect the prevalence. Although each method has it is advantage such as probing depth assess the depth of the periodontal pocket and may represent current disease status [23], CAL and radiograph measure lifetime accumulated past disease [8, 10, 17], bleeding on probing (BOP) indicates the presence of active signs of inflammation [7, 23], there is no consensus regarding the best method used. In the current article, some studies used one method, and other studies combined two or more methods to have more accurate decision.

Many studies have been conducted using different diagnostic classifications regarding periodontitis. The most common classification that used a single criterion was CPI/CPITN (Community Periodontal Index/Community Periodontal Index of Treatment Needs) of which they used PD ≥ 3.5mm as a cut point (13.4%). This classification consumes less time because they only record the upper and lower first molars, the upper right central incisor, and the lower left central incisor [24, 25]. This makes it fast and easy to apply in large samples of people [24, 25]. International uniformity is the most important advantage, but it does not record irreversible changes such as recession or loss of periodontal attachment [26].

Moreover, the 1999 AAP classification (Armitage classification) was not common in these articles. This is not a surprise because it is useful for clinicians [7] and has a little value in establishing case definitions for use in the surveillance of periodontitis worldwide [7].

The most common definition which used two criteria was the CDC/AAP working group, published in 2007, for moderate and severe [7]. They used CAL≥ 4 mm and PD ≥ 5 mm in at least 2 interproximal sites (not on the same tooth) for moderate chronic periodontitis and CAL≥ 6 mm in at least 2 interproximal sites (not on the same tooth) and PD ≥ 5 mm in at least one site for severe chronic periodontitis. Then, they proposed a definition for mild periodontitis in 2012 which was CAL≥ 3 mm and PD ≥ 4 mm in at least 2 interproximal sites (not on the same tooth) or one site with PD ≥ 5 mm [6].

The recent 2017 World Workshop on the Classification of Periodontal and Peri-Implant Diseases and Conditions, copresented by the American Academy of Periodontology (AAP) and the European Federation of Periodontology (EFP) [27, 28], introduced new parameters based on certain articles. However, it is too early to assess the actual effect of this consensus and its acceptance globally.

After results analysis of all variables included in these articles, we found the most present predicators/risk factors associated with chronic periodontitis in periodontal literature were age, gender, and race. In age, as one becomes older, the risk of chronic diseases will increase. The physiological outcome of the ageing process (free radicals), which is the main factor associated with tissue destruction, will result in chronic inflammation [4, 29].

Gender is the second most common variable investigated. Different studies showed that males are more prone to periodontal disease than females, with the assumption of bad oral hygiene and less professional care as the reason for calculus decomposition [30, 31]. There are no enough studies which represent the relationship between race (the third most common variable) and periodontitis.

Cigarette smoking is an important risk factor in the development of inflammatory periodontal diseases [32–34]. It causes harmful effects on gingival tissues, immune responses, and the healing potential of the oral cavity [32, 35, 36]. Diabetes is another commonly investigated factor. Grossi (1994) reported a strong association with diabetes and attachment loss, with an odds ratio of 2.32 (95% CI: 1.17–4.60) [37, 38]. Emrich (1991) investigated type 2 diabetes subjects within the Pima Indian population. They had an increased risk of periodontitis (OR: 2.81, 95% CI: 1.91–4.13), and when bone loss was used to measure periodontal destruction, there was an odds ratio of 3.43 (95% CI: 2.28–5.16) [39].

The least present variables were albumin and creatinine level which may need further investigation in the future. A study by Shimazaki et al. (2013) suggests that there is an inverse relationship between creatinine level and chronic periodontitis. Although some of the biomarkers can be improved by chronic periodontitis treatment, creatinine level depends on renal function [40, 41]. Several studies concluded that there is an inverse relationship between albumin level and periodontitis. Serum albumin level is affected by inflammation and malnutrition; however, other studies found that it difficult to estimate. Although there is a correlation, the relationship between them could not be established [40, 41].

Several clinical examination methods, threshold values, and criteria of chronic periodontitis were used in this research, including measurement of probing depth (PD), clinical attachment loss (CAL), bleeding on probing (BOP), and alveolar bone loss with or without radiographs. Different studies proposed different chronic periodontitis definitions; some used a combination of methods, and some used a single method. Thus, there is a lack of standardization, which leads to difficulty in drawing valid conclusions and serious impairment in accepting the results. Clear definitions of the disease and associated threshold values and criteria should be established worldwide to ensure accurate results in future studies. The most common predictors/confounders associated with the chronic periodontitis were age and gender. However, other variables need more investigation to assess their association with the disease.

## Figures and Tables

**Table 1:** Frequency and percentage of different chronic periodontitis case definitions.

Method	Number of studies(%)
	N=351
*Single criteria*	
CAL	54(15.4)
Radiograph	19(5.4)
PD	110(31.3)
ICD	1(0.3)

*Combined criteria *	
PD+CAL	121(34.5)
Radiograph + PD	5(1.4)
CAL + furcation	1(0.3)
CAL + radiograph	4(1.1)
PD+CAL+BOP	21(6.0)
PD+BOP	7(2.0)
Edema +BOP +PD+ recession +mobility	1(0.3)
PD+ CAL+ Radiograph	4(1.1)
PD+ Furcation	1(0.3)
PD+ Radiograph +BOP	2(0.6)

CAL: clinical attachment level, PD: probing depth, and BOP: bleeding on probing.

**Table 2:** Frequency and percentage of criteria and threshold used in chronic periodontitis case definitions.

Definition	Studies # (%) N=351	Criteria	Studies # (%) N=224
*CAL*			
CAL≥1	15(4.3)	at least 2 sites	6(2.7)
CAL≥2	2(0.6)	at least 3 sites	4(1.8)
CAL≥3	21(6.0)	at least 4 sites	3(1.3)
CAL≥4	11(3.1)	at least 1 site	3(1.3)
CAL≥5	4(1.1)	at least ≥ 50 % sites	1(0.4)
CAL≥6	1(0.3)	CAL ≥3 in at least 2 sites and CAL≥5 in 30%	5(2.2)
*Radiograph*			
Bitewing + periapical	2(0.6)	≥2mm	4(1.8)
Panorama	6(1.7)	≥3mm	2(0.9)
Bitewing	11(3.1)	≥6mm (2 studies at least one side)	2(0.9)
Periapical	1(0.3)	≥20% (one study at least 7 sites)	3(1.3)
		≥50%	1(0.4)
*PD*			
PD≥4	46(13.1)	at least 6 sites	1(0.4)
PD≥3.5	47(13.4)	at least 10 sites	2(0.9)
PD≥3	2(0.6)	at least 1 sites	56(25.0)
PD≥5	12(3.4)	at least 3 sites (one study per quadrant)	3(1.3)
PD≥6	2(0.6)	at least 1 tooth	3(1.3)
PD≥7	1(0.3)	at least 4 sites	2(0.9)
*PD + CAL*			
PD ≥5 CAL≥5	3(0.9)	at least 10% sites (PD+CAL)	1(0.4)
PD ≥5 CAL≥4	26(7.4)	6 teeth with at least 1 site each	1(0.4)
PD ≥5 CAL≥3	6(1.7)	at least 4 sites	2(0.9)
PD ≥3 CAL≥6	1(0.3)	at least 2 sites CAL and 1 site PD	36(16.1)
PD ≥3 CAL≥1	1(0.3)	at least 2 sites	17(7.6)
PD ≥3 CAL≥2	1(0.3)	at least 2 teeth (one study with 3 sites PD and 3 studies with one site)	7(3.1)
PD ≥3 CAL≥3	1(0.3)	at least 3 sites PD and 2 sites CAL	2(0.9)
PD ≥3 CAL≥4	6(1.7)	at least 4 teeth and at least one site each	9(4.0)
PD ≥4 CAL≥3	34(9.7)	at least 6 sites	1(0.4)
PD ≥4 CAL≥2	5(1.4)	at least 6 teeth and at least one site each	1(0.4)
PD ≥4 CAL≥4	14(4.0)	at least 8 teeth	1(0.4)
PD ≥4 CAL≥5	4(1.1)	at least 4 sites (one study 4 sites CAL and one site PD	2(0.9)
PD ≥5 CAL≥6	12(3.4)	at least one posterior tooth	1(0.4)
PD ≥5 CAL≥1	1(0.3)	at least one site	10(4.4)
PD ≥5 CAL≥2	1(0.3)	at least one tooth with at least one site	1(0.4)
PD ≥6 CAL≥4	1(0.3)	at least two molars	1(0.4)
PD ≥6 CAL≥5	3(0.9)	CAL at more than one tooth site and with more than three sites of probing depth	1(0.4)
PD ≥6 CAL≥6	1(0.3)	CAL in at least two sites of different teeth and in at least one proximal site	1(0.4)
		PD in one or more bleeding-positive sites and CAL in two or more sites.	1(0.4)
*PD + Radiograph*			
PD ≥4 Radiograph bitewing	1(0.3)	at least 10 pockets in 10 teeth	1(0.4)
PD ≥5 Radiograph panorama	2(0.6)		
PD ≥5 Radiograph panorama	2(0.6)		
*CAL + furcation*			
CAL ≥1 & furcation	1(0.3)		
*CAL + radiograph*			
CAL ≥1 &Radiograph bitewing	1(0.3)	CAL 30% or more of the sites, and 20>% bone loss as estimated from the radiographs	1(0.4)
CAL ≥2 &Radiograph bitewing	1(0.3)		
CAL ≥4 &Radiograph bitewing	2(0.6)		
*PD+CAL+BOP*			
PD ≥5 CAL≥5 BOP	5(1.4)	10% of teeth with PD or CAL ≥5mmm or 15% of teeth with PD or CAL ≥4mm and 10% of sites with BOP	1(0.4)
PD ≥4 CAL≥3 BOP	8(2.3)	at least 2 teeth and one tooth BOP	1(0.4)
PD ≥4 CAL≥2 BOP	2(0.6)	at least 4 teeth with one or more site	11(4.9)
PD ≥4 CAL≥4 BOP	1(0.3)	at least 5 sites	2(0.9)
PD ≥5 CAL≥3 BOP	4(1.1)	at least 8 sites	1(0.4)
PD ≥6 CAL≥3 BOP	1(0.3)	at least one site	1(0.4)
*PD+BOP*			
PD ≥3 BOP	4(1.1)	at least four teeth	2(0.9)
PD ≥4 BOP	2(0.6)		
PD ≥5 BOP	1(0.3)		
*PD +CAL +Radiograph*			
PD ≥5 CAL≥6 & Radiograph	1(0.3)	at least 2 sites	1(0.4)
PD ≥4 CAL≥4 & Radiograph	2(0.6)	at least 3 sites in at least 3 quadrant	2(0.9)
PD ≥5 CAL≥5 & Radiograph	1(0.3)		
*Edema +BOP +PD+ recession +mobility*			
edema +BOP +PD+ recession +mobility	1(0.3)	If two or more parameters	1(0.4)
*PD+ furcation*			
PD & furcation	1(0.3)	at least 8 teeth	1(0.4)
*PD+Radiograph +BOP*			
PD ≥4 & Radiograph	1(0.3)	10% of teeth with radiograph at least one tooth PD 15% BOP	1(0.4)
PD ≥5 & Radiograph	1(0.3)		

CAL: clinical attachment level, PD: probing depth, and BOP: bleeding on probing.

**Table 3:** Frequency and percentage of chronic periodontitis severity case definitions.

Definition of severity	Studies # (%)
*Severe*	
**CAL:**	
at least 1 site CAL≥6	2(2.1)
at least 2 teeth CAL ≥6 mm	1(1.1)
at least 2 sites CAL≥4	1(1.1)
at least 30% sites CAL≥5	3(3.2)
CAL ≥ 6 mm	2(2.1)
CAL ≥5 mm	15(16.0)
**Radiograph:**	
>50 % of the root	4(4.3)
>33% of the roots, or if angular bony defects/furcation defects degree II and III in more than 3 teeth (molar and premolar regions)	1(1.1)
**PD:**	
PD≥6 mm	4(4.3)
PD of ≥4 mm and at least 10 sites of PD ≥6 mm.	1(1.1)
≥19 sites PD ≥5 mm	1(1.1)
PD 7 to 8 mm	1(1.1)
PD > 7.5 mm	1(1.1)
PD ≥4	1(1.1)
at least 10% of sites with PD≥ 6 mm (moderate to severe )	1(1.1)
**PD + CAL:**	
PD and CAL ≥5 mm	1(1.1)
PD≥4 and CAL≥6	1(1.1)
PD≥5 and CAL≥6	2(2.1)
PD ≥5 and CAL≥4 (moderate or severe)	42(44.7)
at least two sites CAL ≥ 6mm (not on same tooth) and at least one site with PD ≥5mm	1(1.1)
PD≥6 and CAL≥5	1(1.1)
PD and CAL ≥5mm	2(2.1)
PD and CAL≥6 mm	1(1.1)
**CAL or PD with radiograph:**	
PD≥5 and radiographic panorama ≥30	1(1.1)
CAL >6 mm, grade II and/or III furcation, possible tooth mobility class II or III	1(1.1)
**PD + CAL+ BOP:**	
PD≥5, CAL≥6 and BOP	1(1.1)
PD≥5, CAL≥6 BOP≥30	1(1.1)
Total	94(100)
*Moderate*	
**CAL:**	
CAL 3–4 mm	15(42.9)
CAL 3 to 6 mm CAL	1(2.9)
3 sites with CAL ≥4 mm	1(2.9)
**Radiograph:**	
20-30% of the root	1(2.9)
>2 mm not exceeding one-third of the roots, or if angular bony defects/furcation defects degree II and III in 2-3 teeth (molar and premolar regions)	1(2.9)
**PD:**	
PD 4-5 mm	1(2.9)
PD with ≥4 mm and a maximum of 9 sites with PPD of ≥6 mm	1(2.9)
≥7 to 18 sites PD ≥5 mm	1(2.9)
PD ≥3	1(2.9)
PD 5.5 and 7.5 mm	1(2.9)
PD 5 to 8mm	1(2.9)
**PD + CAL:**	
PD≥4 and CAL≥4	1(2.9)
PD ≥5 and CAL≥4	1(2.9)
PD >5 mm and CAL = 3–4 mm	1(2.9)
PD and CAL≥3 and <5mm	2(5.7)
PD≥4 and CAL≥4 mm	1(2.9)
at least one site with both ≥ 4 mm and PD of ≥4 mm	1(2.9)
**CAL or PD with radiograph:**	
CAL 4–6 mm, grade I and/or II furcation, and possible tooth mobility class I	1(2.9)
**PD + CAL+ BOP**	
PD≥5, CAL≥4 and BOP	1(2.9)
PD≥5, CAL≥3 and BOP≥30	1(2.9)
Total	35(100)
*Mild*	
**CAL:**	
CAL 4–5 mm	1(4.0)
at least 2 teeth CAL ≥1 mm	1(4.0)
at least at one site CAL < 3 mm	1(4.0)
CAL of 1–2 mm	15(60.0)
**PD:**	
mild or no disease with PPD of ≤4 mm	1(4.0)
≥3 to 6 sites that are PD ≥5 mm	1(4.0)
PD 4 to 5mm	1(4.0)
PD 3.5 and 5.5 mm	1(4.0)
at least 10% of sites with PD of 5 mm	1(4.0)
**PD + CAL**	
PD > 5 mm and CAL = 1–2	1(4.0)
**CAL or PD with radiograph**	
CAL ≤4 mm and possible class I furcation invasion areas	1(4.0)
Total	25(100)

CAL: clinical attachment level, PD: probing depth, and BOP: bleeding on probing.

## Additional Points

*Scientific Rationale for the Study*. Case definitions and criteria of periodontal diseases are not yet consistent worldwide. This can affect the accuracy of any comparison made between two studies. Moreover, several articles have shown that there are various factors associated with periodontitis. Thus, little is known about the common case definitions and confounders of chronic periodontitis in epidemiological literature.* Principal Findings. *The included studies used different periodontitis definitions and criteria. Some used combinations parameters, and some used a single parameter, which led to difficulty in drawing valid conclusions as well as serious impairment in accepting the results.* Practical Implications. *A clear definition of the disease and associated parameters should be established worldwide for solid results in future studies. In addition, several variables need more investigation to assess their association with the disease.

## Supplementary Materials

Supplementary Materials S1 Text: list of included articles categorized based on each criterion used in chronic periodontitis case definitions: CAL: clinical attachment level, PD: Probing depth, and BOP: bleeding on probing.
