# Supplementary material for: Chronic Periodontitis Case Definitions and Confounders in Periodontal Research: A Systematic Assessment
Source: Biomed Res Int. 2018 Nov 28;2018:4578782. doi: 10.1155/2018/4578782 (PMC6304204; doi:10.1155/2018/4578782)
Supplement: Supplementary Materials — S1 Text: list of included articles categorized based on each criterion used in chronic periodontitis case definitions: CAL: clinical attachment level, PD: Probing depth, and BOP: bleeding on probing. [file 4578782.f1.docx]

**Supplementary Appendix:**

*Supplementary Appendix : CAL*

Adegboye, A. R., Christensen, L. B., Holm-Pedersen, P., Avlund, K., Boucher, B. J. & Heitmann, B. L. (2012) Intake of dairy products in relation to periodontitis in older Danish adults. *Nutrients* **4,** 1219-1229. doi:10.3390/nu4091219.

Ansari Moghadam, S., Abdollahi, Z., Risbaf Fakour, S., Ansari Moghaddam, A., Kiany, F. & Damani, N. (2015) The Relationship Between Periodontal Disease and Public Health: A Population-Based Study. *Glob J Health Sci* **8,** 110-115. doi:10.5539/gjhs.v8n7p110.

Beck, J. D., Koch, G. G. & Offenbacher, S. (1994) Attachment loss trends over 3 years in community-dwelling older adults. *J Periodontol* **65,** 737-743. doi:10.1902/jop.1994.65.8.737.

Beck, J. D., Sharp, T., Koch, G. G. & Offenbacher, S. (1997) A study of attachment loss patterns in survivor teeth at 18 months, 36 months and 5 years in community-dwelling older adults. *J Periodontal Res* **32,** 497-505.

Borges-Yanez, S. A., Irigoyen-Camacho, M. E. & Maupome, G. (2006) Risk factors and prevalence of periodontitis in community-dwelling elders in Mexico. *J Clin Periodontol* **33,** 184-194. doi:10.1111/j.1600-051X.2006.00897.x.

Bostanci, V., Toker, H., Senel, S. & Sahin, S. (2014) Prevalence of periodontal disease in patients with Familial Mediterranean Fever: a cohort study from central Turkey. *Quintessence Int* **45,** 743-748. doi:10.3290/j.qi.a32442.

Bourgeois, D., Bouchard, P. & Mattout, C. (2007) Epidemiology of periodontal status in dentate adults in France, 2002-2003. *J Periodontal Res* **42,** 219-227. doi:10.1111/j.1600-0765.2006.00936.x.

Bourgeois, D., Hescot, P. & Doury, J. (1997) Periodontal conditions in 35-44-yr-old adults in France, 1993. *J Periodontal Res* **32,** 570-574.

Bourgeois, D. M., Doury, J. & Hescot, P. (1999) Periodontal conditions in 65-74 year old adults in France, 1995. *Int Dent J* **49,** 182-186.

Brito, F., de Barros, F. C., Zaltman, C., Carvalho, A. T., Carneiro, A. J., Fischer, R. G., Gustafsson, A. & Figueredo, C. M. (2008) Prevalence of periodontitis and DMFT index in patients with Crohn's disease and ulcerative colitis. *J Clin Periodontol* **35,** 555-560. doi:10.1111/j.1600-051X.2008.01231.x.

Castrejon-Perez, R. C., Jimenez-Corona, A., Bernabe, E., Villa-Romero, A. R., Arrive, E., Dartigues, J. F., Gutierrez-Robledo, L. M. & Borges-Yanez, S. A. (2017) Oral Disease and 3-Year Incidence of Frailty in Mexican Older Adults. *J Gerontol A Biol Sci Med Sci* **72,** 951-957. doi:10.1093/gerona/glw201.

Cho-Yan Lee, J., Mattheos, N., Nixon, K. C. & Ivanovski, S. (2012) Residual periodontal pockets are a risk indicator for peri-implantitis in patients treated for periodontitis. *Clin Oral Implants Res* **23,** 325-333. doi:10.1111/j.1600-0501.2011.02264.x.

Choi, I. A., Kim, J. H., Kim, Y. M., Lee, J. Y., Kim, K. H., Lee, E. Y., Lee, E. B., Lee, Y. M. & Song, Y. W. (2016) Periodontitis is associated with rheumatoid arthritis: a study with longstanding rheumatoid arthritis patients in Korea. *Korean J Intern Med* **31,** 977-986. doi:10.3904/kjim.2015.202.

Choi, Y. H., McKeown, R. E., Mayer-Davis, E. J., Liese, A. D., Song, K. B. & Merchant, A. T. (2011) Association between periodontitis and impaired fasting glucose and diabetes. *Diabetes Care* **34,** 381-386. doi:10.2337/dc10-1354.

Clerehugh, V., Lennon, M. A. & Worthington, H. V. (1990) 5-year results of a longitudinal study of early periodontitis in 14- to 19-year-old adolescents. *J Clin Periodontol* **17,** 702-708.

Collins, J., Carpio, A. M., Bobadilla, M., Reyes, R., Guzman, I., Martinez, B. & Gamonal, J. (2005) Prevalence of clinical attachment loss in adolescents in Santo Domingo, Dominican Republic. *J Periodontol* **76,** 1450-1454. doi:10.1902/jop.2005.76.9.1450.

Costa, F. O., Cota, L. O., Lages, E. J., Cyrino, R. M., Oliveira, A. M., Oliveira, P. A. & Cortelli, J. R. (2013) Associations of duration of smoking cessation and cumulative smoking exposure with periodontitis. *J Oral Sci* **55,** 245-253.

Dalla Vecchia, C. F., Susin, C., Rosing, C. K., Oppermann, R. V. & Albandar, J. M. (2005) Overweight and obesity as risk indicators for periodontitis in adults. *J Periodontol* **76,** 1721-1728. doi:10.1902/jop.2005.76.10.1721.

Deppe, H., Mucke, T., Wagenpfeil, S., Kesting, M., Karl, J., Noe, S. & Sculean, A. (2015) Are selected IL-1 polymorphisms and selected subgingival microorganisms significantly associated to periodontitis in type 2 diabetes patients? a clinical study. *BMC Oral Health* **15,** 143. doi:10.1186/s12903-015-0132-5.

Fernandes, J. K., Wiegand, R. E., Salinas, C. F., Grossi, S. G., Sanders, J. J., Lopes-Virella, M. F. & Slate, E. H. (2009) Periodontal disease status in gullah african americans with type 2 diabetes living in South Carolina. *J Periodontol* **80,** 1062-1068. doi:10.1902/jop.2009.080486.

Flemmig, T. F., Petersilka, G., Volp, A., Gravemeier, M., Zilly, M., Mross, D., Prior, K., Yamamoto, J. & Beikler, T. (2011) Efficacy and safety of adjunctive local moxifloxacin delivery in the treatment of periodontitis. *J Periodontol* **82,** 96-105. doi:10.1902/jop.2010.100124.

Friedrich, N., Kocher, T., Wallaschofski, H., Schwahn, C., Ludemann, J., Kerner, W. & Volzke, H. (2008) Inverse association between periodontitis and respiratory allergies in patients with type 1 diabetes mellitus. *J Clin Periodontol* **35,** 305-310. doi:10.1111/j.1600-051X.2008.01200.x.

Friedrich, N., Volzke, H., Schwahn, C., Kramer, A., Junger, M., Schafer, T., John, U. & Kocher, T. (2006) Inverse association between periodontitis and respiratory allergies. *Clin Exp Allergy* **36,** 495-502. doi:10.1111/j.1365-2222.2006.02455.x.

Gamonal, J., Mendoza, C., Espinoza, I., Munoz, A., Urzua, I., Aranda, W., Carvajal, P. & Arteaga, O. (2010) Clinical attachment loss in Chilean adult population: First Chilean National Dental Examination Survey. *J Periodontol* **81,** 1403-1410. doi:10.1902/jop.2010.100148.

Gay, I. C., Tran, D. T., Cavender, A. C., Weltman, R., Chang, J., Luckenbach, E. & Tribble, G. D. (2014) The effect of periodontal therapy on glycaemic control in a Hispanic population with type 2 diabetes: a randomized controlled trial. *J Clin Periodontol* **41,** 673-680. doi:10.1111/jcpe.12268.

Ha, J. E., Jun, J. K., Ko, H. J., Paik, D. I. & Bae, K. H. (2014) Association between periodontitis and preeclampsia in never-smokers: a prospective study. *J Clin Periodontol* **41,** 869-874. doi:10.1111/jcpe.12281.

Hach, M., Holm-Pedersen, P., Adegboye, A. R. & Avlund, K. (2015) The effect of alcohol consumption on periodontitis in older Danes. *Int J Dent Hyg* **13,** 261-267. doi:10.1111/idh.12121.

Harikishan, G., Triveni, V. S. & Sai Sujay, G. S. (2015) Evaluation of clinical parameters to select high prevalence populations for periodontal disease: A cross-sectional study. *J Pharm Bioallied Sci* **7,** S623-627. doi:10.4103/0975-7406.163573.

Harks, I., Koch, R., Eickholz, P., Hoffmann, T., Kim, T. S., Kocher, T., Meyle, J., Kaner, D., Schlagenhauf, U., Doering, S., Holtfreter, B., Gravemeier, M., Harmsen, D. & Ehmke, B. (2015) Is progression of periodontitis relevantly influenced by systemic antibiotics? A clinical randomized trial. *J Clin Periodontol* **42,** 832-842. doi:10.1111/jcpe.12441.

Hayman, L., Steffen, M. J., Stevens, J., Badger, E., Tempro, P., Fuller, B., McGuire, A., Al-Sabbagh, M., Thomas, M. V. & Ebersole, J. L. (2011) Smoking and periodontal disease: discrimination of antibody responses to pathogenic and commensal oral bacteria. *Clin Exp Immunol* **164,** 118-126. doi:10.1111/j.1365-2249.2010.04314.x.

Herrera, D., Contreras, A., Gamonal, J., Oteo, A., Jaramillo, A., Silva, N., Sanz, M., Botero, J. E. & Leon, R. (2008) Subgingival microbial profiles in chronic periodontitis patients from Chile, Colombia and Spain. *J Clin Periodontol* **35,** 106-113. doi:10.1111/j.1600-051X.2007.01170.x.

Hirotomi, T., Yoshihara, A., Ogawa, H. & Miyazaki, H. (2010) Tooth-related risk factors for periodontal disease in community-dwelling elderly people. *J Clin Periodontol* **37,** 494-500. doi:10.1111/j.1600-051X.2010.01565.x.

Hugoson, A. & Norderyd, O. (2008) Has the prevalence of periodontitis changed during the last 30 years? *J Clin Periodontol* **35,** 338-345. doi:10.1111/j.1600-051X.2008.01279.x.

Iida, H., Kumar, J. V., Kopycka-Kedzierawski, D. T. & Billings, R. J. (2009) Effect of tobacco smoke on the oral health of U.S. women of childbearing age. *J Public Health Dent* **69,** 231-241. doi:10.1111/j.1752-7325.2009.00128.x.

Kebede, T. G., Holtfreter, B., Kocher, T., Meisel, P., Dietrich, T., Biffar, R., Dorr, M., Volzke, H. & Pink, C. (2017) Association of Periodontal Destruction and Diabetes with Mortality. *J Dent Res* **96,** 56-63. doi:10.1177/0022034516668839.

Kshirsagar, A. V., Craig, R. G., Moss, K. L., Beck, J. D., Offenbacher, S., Kotanko, P., Klemmer, P. J., Yoshino, M., Levin, N. W., Yip, J. K., Almas, K., Lupovici, E. M., Usvyat, L. A. & Falk, R. J. (2009) Periodontal disease adversely affects the survival of patients with end-stage renal disease. *Kidney Int* **75,** 746-751. doi:10.1038/ki.2008.660.

Lalla, E., Cheng, B., Lal, S., Tucker, S., Greenberg, E., Goland, R. & Lamster, I. B. (2006) Periodontal changes in children and adolescents with diabetes: a case-control study. *Diabetes Care* **29,** 295-299.

Matevosyan, N. R. (2011) Periodontal disease and perinatal outcomes. *Arch Gynecol Obstet* **283,** 675-686. doi:10.1007/s00404-010-1774-9.

Minaya-Sanchez, M., Medina-Solis, C. E., Maupome, G., Vallejos-Sanchez, A. A., Casanova-Rosado, J. F. & Marquez-Corona Mde, L. (2007) Prevalence of and risk indicators for chronic periodontitis in males from Campeche, Mexico. *Rev Salud Publica (Bogota)* **9,** 388-398.

Nabet, C., Lelong, N., Colombier, M. L., Sixou, M., Musset, A. M., Goffinet, F. & Kaminski, M. (2010) Maternal periodontitis and the causes of preterm birth: the case-control Epipap study. *J Clin Periodontol* **37,** 37-45. doi:10.1111/j.1600-051X.2009.01503.x.

Pei, X., Ouyang, X., He, L., Cao, C., Luan, Q. & Suda, R. (2015) A 4-year prospective study of the progression of periodontal disease in a rural Chinese population. *J Dent* **43,** 192-200. doi:10.1016/j.jdent.2014.12.008.

Peter, K. P., Mute, B. R., Pitale, U. M., Shetty, S., Hc, S. & Satpute, P. S. (2014) Prevalence of periodontal disease and characterization of its extent and severity in an adult population - an observational study. *J Clin Diagn Res* **8,** ZC04-07. doi:10.7860/jcdr/2014/8684.5231.

Rakoto-Alson, S., Tenenbaum, H. & Davideau, J. L. (2010) Periodontal diseases, preterm births, and low birth weight: findings from a homogeneous cohort of women in Madagascar. *J Periodontol* **81,** 205-213. doi:10.1902/jop.2009.090351.

Reichert, S., Schulz, S., Benten, A. C., Lutze, A., Seifert, T., Schlitt, M., Werdan, K., Hofmann, B., Wienke, A., Schaller, H. G. & Schlitt, A. (2016) Periodontal conditions and incidence of new cardiovascular events among patients with coronary vascular disease. *J Clin Periodontol* **43,** 918-925. doi:10.1111/jcpe.12611.

Rheu, G. B., Ji, S., Ryu, J. J., Lee, J. B., Shin, C., Lee, J. Y., Huh, J. B. & Shin, S. W. (2011) Risk assessment for clinical attachment loss of periodontal tissue in Korean adults. *J Adv Prosthodont* **3,** 25-32. doi:10.4047/jap.2011.3.1.25.

Sabbah, W., Tsakos, G., Chandola, T., Newton, T., Kawachi, I., Sheiham, A., Marmot, M. G. & Watt, R. G. (2011) The relationship between social network, social support and periodontal disease among older Americans. *J Clin Periodontol* **38,** 547-552. doi:10.1111/j.1600-051X.2011.01713.x.

Sakalauskiene, J., Kubilius, R., Gleiznys, A., Vitkauskiene, A., Ivanauskiene, E. & Saferis, V. (2014) Relationship of clinical and microbiological variables in patients with type 1 diabetes mellitus and periodontitis. *Med Sci Monit* **20,** 1871-1877. doi:10.12659/msm.890879.

Selikowitz, H. S. (1987) The relationship between periodontal conditions and perceptions of periodontal health among Pakistani immigrants in Norway. *J Clin Periodontol* **14,** 340-344.

Selikowitz, H. S. & Gjermo, P. (1985) Periodontal conditions, remaining teeth and oral hygiene habits in a group of Vietnamese refugees in Norway. *J Clin Periodontol* **12,** 42-50.

Silva, A. M., Vargas, A. M., Ferreira, E. F. & de Abreu, M. H. (2010) Periodontitis in individuals with diabetes treated in the public health system of Belo Horizonte, Brazil. *Rev Bras Epidemiol* **13,** 118-125.

Smith, G. L., Cross, D. L. & Wray, D. (1995) Comparison of periodontal disease in HIV seropositive subjects and controls (I). Clinical features. *J Clin Periodontol* **22,** 558-568.

Sperr, M., Kundi, M., Tursic, V., Bristela, M., Moritz, A., Andrukhov, O., Rausch-Fan, X. & Sperr, W. R. (2017) Prevalence of Comorbidities in Periodontitis Patients Compared to the General Austrian Population. *J Periodontol***,** 1-13. doi:10.1902/jop.2017.170333.

Susin, C., Haas, A. N., Valle, P. M., Oppermann, R. V. & Albandar, J. M. (2011) Prevalence and risk indicators for chronic periodontitis in adolescents and young adults in south Brazil. *J Clin Periodontol* **38,** 326-333. doi:10.1111/j.1600-051X.2011.01699.x.

Tarannum, F. & Faizuddin, M. (2007) Effect of periodontal therapy on pregnancy outcome in women affected by periodontitis. *J Periodontol* **78,** 2095-2103. doi:10.1902/jop.2007.060388.

Vidal, F., Figueredo, C. M., Cordovil, I. & Fischer, R. G. (2011) Higher prevalence of periodontitis in patients with refractory arterial hypertension: a case-control study. *Oral Dis* **17,** 560-563. doi:10.1111/j.1601-0825.2011.01800.x.

Wolff, B., Berger, T., Frese, C., Max, R., Blank, N., Lorenz, H. M. & Wolff, D. (2014) Oral status in patients with early rheumatoid arthritis: a prospective, case-control study. *Rheumatology (Oxford)* **53,** 526-531. doi:10.1093/rheumatology/ket362.

*Supplementary Appendix : Radiograph*

Aass, A. M., Tollefsen, T. & Gjermo, P. (1994) A Cohort Study of Radiographic Alveolar Bone Loss during Adolescence. *Journal of Clinical Periodontology* **21,** 133-138.

Blankenstein, R., Murray, J. J. & Lind, O. P. (1978) Prevalence of Chronic Periodontitis in 13-15-Year-Old Children - Radiographic Study. *Journal of Clinical Periodontology* **5,** 285-292.

Edman, K., Ohrn, K., Nordstrom, B., Holmlund, A. & Hellberg, D. (2015) Trends over 30 years in the prevalence and severity of alveolar bone loss and the influence of smoking and socio-economic factors--based on epidemiological surveys in Sweden 1983-2013. *Int J Dent Hyg* **13,** 283-291. doi:10.1111/idh.12164.

Haber, J. & Kent, R. L. (1992) Cigarette smoking in a periodontal practice. *J Periodontol* **63,** 100-106. doi:10.1902/jop.1992.63.2.100.

Hansen, B. F., Gjermo, P. & Bergwitzlarsen, K. R. (1984) Periodontal Bone Loss in 15-Year-Old Norwegians. *Journal of Clinical Periodontology* **11,** 125-131.

Haubek, D., Ennibi, O. K., Poulsen, K., Vaeth, M., Poulsen, S. & Kilian, M. (2008) Risk of aggressive periodontitis in adolescent carriers of the JP2 clone of Aggregatibacter (Actinobacillus) actinomycetemcomitans in Morocco: a prospective longitudinal cohort study. *Lancet* **371,** 237-242.

Hull, P. S., Hillam, D. G. & Beal, J. F. (1975) A Radiographic Study of the Prevalence of Chronic Periodontitis in 14 Year Old English School Children. *Journal of Clinical Periodontology* **2,** 203-210.

Jimenez, M., Giovannucci, E., Krall Kaye, E., Joshipura, K. J. & Dietrich, T. (2014) Predicted vitamin D status and incidence of tooth loss and periodontitis. *Public Health Nutr* **17,** 844-852. doi:10.1017/s1368980013000177.

Jimenez, M., Krall, E. A., Garcia, R. I., Vokonas, P. S. & Dietrich, T. (2009) Periodontitis and incidence of cerebrovascular disease in men. *Ann Neurol* **66,** 505-512. doi:10.1002/ana.21742.

Joseph, B. K., Kullman, L. & Sharma, P. N. (2016) The oral-systemic disease connection: a retrospective study. *Clin Oral Investig* **20,** 2267-2273. doi:10.1007/s00784-016-1725-3.

Laine, M. L., Morre, S. A., Murillo, L. S., van Winkelhoff, A. J. & Pena, A. S. (2005) CD14 and TLR4 gene polymorphisms in adult periodontitis. *J Dent Res* **84,** 1042-1046. doi:10.1177/154405910508401114.

Latcham, N. L., Powell, R. N., Jago, J. D., Seymour, G. J. & Aitken, J. F. (1983) A Radiographic Study of Chronic Periodontitis in 15 Year Old Queensland Children. *Journal of Clinical Periodontology* **10,** 37-45.

Leuckfeld, I., Obregon-Whittle, M. V., Lund, M. B., Geiran, O., Bjortuft, O. & Olsen, I. (2008) Severe chronic obstructive pulmonary disease: association with marginal bone loss in periodontitis. *Respir Med* **102,** 488-494. doi:10.1016/j.rmed.2007.12.001.

Maffei, G., Brouwer, N., Dolman, K. M., van der Velden, U., Roos, D. & Loos, B. G. (2005) Plasma levels of mannan-binding lectin in relation to periodontitis and smoking. *J Periodontol* **76,** 1881-1889. doi:10.1902/jop.2005.76.11.1881.

Moergel, M., Kammerer, P., Kasaj, A., Armouti, E., Alshihri, A., Weyer, V. & Al-Nawas, B. (2013) Chronic periodontitis and its possible association with oral squamous cell carcinoma - a retrospective case control study. *Head Face Med* **9,** 39. doi:10.1186/1746-160x-9-39.

Natto, Z. S. & Al-Zahrani, M. S. (2010) Periodontal bone loss and self-reported medical conditions in a dental school patient population. *J Int Acad Periodontol* **12,** 104-109.

Nesbitt, M. J., Reynolds, M. A., Shiau, H., Choe, K., Simonsick, E. M. & Ferrucci, L. (2010) Association of periodontitis and metabolic syndrome in the Baltimore Longitudinal Study of Aging. *Aging Clin Exp Res* **22,** 238-242.

Zadik, Y., Bechor, R., Galor, S., Justo, D. & Heruti, R. J. (2009) Erectile dysfunction might be associated with chronic periodontal disease: two ends of the cardiovascular spectrum. *J Sex Med* **6,** 1111-1116. doi:10.1111/j.1743-6109.2008.01141.x.

Zadik, Y., Bechor, R., Galor, S. & Levin, L. (2010) Periodontal disease might be associated even with impaired fasting glucose. *Br Dent J* **208,** E20. doi:10.1038/sj.bdj.2010.291.

*Supplementary Appendix : PD*

Adegbembo, A. O. & el-Nadeef, M. A. (1995) National survey of periodontal status and treatment need among Nigerians. *International dental journal* **45,** 197-203.

Ahn, Y. B., Shin, M. S., Byun, J. S. & Kim, H. D. (2015) The association of hypertension with periodontitis is highlighted in female. *J Clin Periodontol* **42,** 998-1005.

Al-Sudani, F. Y., Vehkalahti, M. M. & Suominen, A. L. (2015) The association between current unemployment and clinically determined poor oral health. *Community Dent Oral Epidemiol* **43,** 325-337. doi:10.1111/cdoe.12157.

Ali, R. W., Velcescu, C., Jivanescu, M. C., Lofthus, B. & Skaug, N. (1996) Prevalence of 6 putative periodontal pathogens in subgingival plaque samples from Romanian adult periodontitis patients. *Journal of Clinical Periodontology* **23,** 133-139.

Almerich-Silla, J. M., Alminana-Pastor, P. J., Boronat-Catala, M., Bellot-Arcis, C. & Montiel-Company, J. M. (2017) Socioeconomic factors and severity of periodontal disease in adults (35-44 years). A cross sectional study. *J Clin Exp Dent* **9,** e988-e994. doi:10.4317/jced.54033.

Amarasena, G. & Ekanayake, L. (2010) Periodontal status and associated factors in 15-year-old Sri Lankans. *J Investig Clin Dent* **1,** 74-78. doi:10.1111/j.2041-1626.2010.00025.x.

Anil, S., Hari, S. & Vijayakumar, T. (1990) Periodontal conditions of a selected population in Trivandrum District, Kerala. *Community Dent Oral Epidemiol* **18,** 325.

Antal, M., Braunitzer, G., Mattheos, N., Gyulai, R. & Nagy, K. (2014) Smoking as a permissive factor of periodontal disease in psoriasis. *PLoS One* **9,** e92333. doi:10.1371/journal.pone.0092333.

Ardila, C. M., Fernandez, N. & Guzman, I. C. (2010a) Antimicrobial susceptibility of moxifloxacin against gram-negative enteric rods. *J Periodontol* **81,** 292-299.

Ardila, C. M., Granada, M. I. & Guzman, I. C. (2010b) Antibiotic resistance of subgingival species in chronic periodontitis patients. *J Periodontal Res* **45,** 557-563. doi:10.1111/j.1600-0765.2010.01274.x.

Avlund, K., Schultz-Larsen, K., Krustrup, U., Christiansen, N. & Holm-Pedersen, P. (2009) Effect of inflammation in the periodontium in early old age on mortality at 21-year follow-up. *J Am Geriatr Soc* **57,** 1206-1212. doi:10.1111/j.1532-5415.2009.02328.x.

Bergstrom, J. (1989) Cigarette smoking as risk factor in chronic periodontal disease. *Community Dent Oral Epidemiol* **17,** 245-247.

Bokhari, S. A., Suhail, A. M., Malik, A. R. & Imran, M. F. (2015) Periodontal disease status and associated risk factors in patients attending a Dental Teaching Hospital in Rawalpindi, Pakistan. *J Indian Soc Periodontol* **19,** 678-682.

Buhlin, K., Hultin, M., Norderyd, O., Persson, L., Pockley, A. G., Pussinen, P. J., Rabe, P., Klinge, B. & Gustafsson, A. (2009) Periodontal treatment influences risk markers for atherosclerosis in patients with severe periodontitis. *Atherosclerosis* **206,** 518-522. doi:10.1016/j.atherosclerosis.2009.03.035.

C.J., H. & Corbet, E. F. (1990) Relationship between periodontal parameters and CPITN scores. *- Community Dent Oral Epidemiol* **18,** 322-323.

Chung, J. H., Hwang, H. J., Kim, S. H. & Kim, T. H. (2016) Associations Between Periodontitis and Chronic Obstructive Pulmonary Disease: The 2010 to 2012 Korean National Health and Nutrition Examination Survey. *J Periodontol* **87,** 864-871. doi:10.1902/jop.2016.150682.

de Castilhos, E. D., Horta, B. L., Gigante, D. P., Demarco, F. F., Peres, K. G. & Peres, M. A. (2012) Association between obesity and periodontal disease in young adults: a population-based birth cohort. *J Clin Periodontol* **39,** 717-724. doi:10.1111/j.1600-051X.2012.01906.x.

Delgado-Angulo, E. K., Sabbah, W., Suominen, A. L., Vehkalahti, M. M., Knuuttila, M., Partonen, T., Nordblad, A., Sheiham, A., Watt, R. G. & Tsakos, G. (2015) The association of depression and anxiety with dental caries and periodontal disease among Finnish adults. *Community Dent Oral Epidemiol* **43,** 540-549. doi:10.1111/cdoe.12179.

Deng, T., Wang, L., Lv, J., Pang, J., Liu, B., Du, Y. & Ke , J. (2011) Association of three bacterial species and periodontal status in Chinese adults. *J Clin Microbiol* **49,** 184-188.

Diamanti-Kipioti, A., Papapanou, P. N., Moraitaki-Tsami, A., Lindhe, J. & Mitsis, F. (1993) Comparative estimation of periodontal conditions by means of different index systems. *J Clin Periodontol* **20,** 656-661.

El-Qaderi, S. S. & Quteish Ta'ani, D. (2004) Assessment of periodontal knowledge and periodontal status of an adult population in Jordan. *Int J Dent Hyg* **2,** 132-136. doi:10.1111/j.1601-5037.2004.00080.x.

Flores-de-Jacoby, L., Bruchmann, S., Mengel, R. & Zafiropoulos, G. G. (1991) Periodontal conditions in Rio de Janeiro City (Brazil) using the CPITN. *Community Dent Oral Epidemiol* **19,** 127-128.

Gjermo, P., Bellini, H. T. & Marcos, B. (1983) Application of the Community Periodontal Index of Treatment Needs (CPITN) in a population of young Brazilians. *Community Dent Oral Epidemiol* **11,** 342-346.

Gopalakrishnapillai, A. C., Iyer, R. R. & Kalantharakath, T. (2012) Prevalence of periodontal disease among inpatients in a psychiatric hospital in India. *Spec Care Dentist* **32,** 196-204. doi:10.1111/j.1754-4505.2012.00271.x.

Goultschin, J., Cohen, H. D., Donchin, M., Brayer, L. & Soskolne, W. A. (1990) Association of smoking with periodontal treatment needs. *J Periodontol* **61,** 364-367. doi:10.1902/jop.1990.61.6.364.

Grytten, J., Steele, L. & Holst, D. (1991) Relationship between Number of Teeth and Periodontal Pockets. *Community Dentistry and Oral Epidemiology* **19,** 147-150.

Gursoy, U. K., Liukkonen, J., Jula, A., Huumonen, S., Suominen, A. L., Puukka, P. & Kononen, E. (2016) Associations Between Salivary Bone Metabolism Markers and Periodontal Breakdown. *J Periodontol* **87,** 367-375.

Han, D. H., Khang, Y. H., Jung-Choi, K. & Lim, S. (2013) Association between shift work and periodontal health in a representative sample of an Asian population. *Scand J Work Environ Health* **39,** 559-567. doi:10.5271/sjweh.3370.

Han, D. H., Lim, S., Paek, D. & Kim, H. D. (2012) Periodontitis could be related factors on metabolic syndrome among Koreans: a case-control study. *J Clin Periodontol* **39,** 30-37. doi:10.1111/j.1600-051X.2011.01806.x.

Han, D. H., Lim, S. Y., Sun, B. C., Janket, S. J., Kim, J. B., Paik, D. I., Paek, D. & Kim, H. D. (2009) Mercury exposure and periodontitis among a Korean population: the Shiwha-Banwol environmental health study. *J Periodontol* **80,** 1928-1936. doi:10.1902/jop.2009.090293.

Han, D. H., Lim, S. Y., Sun, B. C., Paek, D. & Kim, H. D. (2010a) The association of metabolic syndrome with periodontal disease is confounded by age and smoking in a Korean population: the Shiwha-Banwol Environmental Health Study. *J Clin Periodontol* **37,** 609-616. doi:10.1111/j.1600-051X.2010.01580.x.

Han, D. H., Lim, S. Y., Sun, B. C., Paek, D. M. & Kim, H. D. (2010b) Visceral fat area-defined obesity and periodontitis among Koreans. *J Clin Periodontol* **37,** 172-179. doi:10.1111/j.1600-051X.2009.01515.x.

Han, K., Hwang, E. & Park, J. B. (2016a) Association between Consumption of Coffee and the Prevalence of Periodontitis: The 2008-2010 Korea National Health and Nutrition Examination Survey. *PLoS One* **11,** e0158845. doi:10.1371/journal.pone.0158845.

Han, K., Hwang, E. & Park, J. B. (2016b) Excessive Consumption of Green Tea as a Risk Factor for Periodontal Disease among Korean Adults. *Nutrients* **8**. doi:10.3390/nu8070408.

Han, K. & Park, J. B. (2017) Age threshold for moderate and severe periodontitis among Korean adults without diabetes mellitus, hypertension, metabolic syndrome, and/or obesity. *Medicine (Baltimore)* **96,** e7835. doi:10.1097/md.0000000000007835.

Hohlfeld, M. & Bernimoulin, J. P. (1993) Application of the community periodontal index of treatment needs (CPITN) in a group of 45-54-year-old German factory workers. *J Clin Periodontol* **20,** 551-556.

Holmlund, A., Holm, G. & Lind, L. (2006) Severity of periodontal disease and number of remaining teeth are related to the prevalence of myocardial infarction and hypertension in a study based on 4,254 subjects. *J Periodontol* **77,** 1173-1178. doi:10.1902/jop.2006.050233.

Hong, M., Kim, H. Y., Seok, H., Yeo, C. D., Kim, Y. S., Song, J. Y., Lee, Y. B., Lee, D. H., Lee, J. I., Lee, T. K., Ahn, H. S., Ko, Y. H., Jeong, S. C., Chae, H. S. & Sohn, T. S. (2016) Prevalence and risk factors of periodontitis among adults with or without diabetes mellitus. *Korean J Intern Med* **31,** 910-919. doi:10.3904/kjim.2016.031.

Hoover, J. N. & Tynan, J. J. (1986) Application of the Community Periodontal Index of Treatment Needs (CPITN) in a group of Canadian adults. *Oral Health* **76,** 13-15.

Hopcraft, M. S., Morgan, M. V., Satur, J. G., Wright, F. A. & Darby, I. B. (2012) Oral hygiene and periodontal disease in Victorian nursing homes. *Gerodontology* **29,** e220-228. doi:10.1111/j.1741-2358.2010.00448.x.

Hugoson, A., Koch, G., Bergendal, T., Hallonsten, A. L., Slotte, C., Thorstensson, B. & Thorstensson, H. (1995) Oral health of individuals aged 3-80 years in Jonkoping, Sweden in 1973, 1983, and 1993. II. Review of clinical and radiographic findings. *Swed Dent J* **19,** 243-260.

Hugoson, A., Laurell, L. & Lundgren, D. (1992) Frequency-Distribution of Individuals Aged 20-70 Years According to Severity of Periodontal-Disease Experience in 1973 and 1983. *Journal of Clinical Periodontology* **19,** 227-232.

Ide, R., Hoshuyama, T., Wilson, D., Takahashi, K. & Higashi, T. (2011) Periodontal disease and incident diabetes: a seven-year study. *J Dent Res* **90,** 41-46.

Iida, H., Kumar, J. V., Kopycka-Kedzierawski, D. T. & Billings, R. J. (2009) Effect of tobacco smoke on the oral health of U.S. women of childbearing age. *J Public Health Dent* **69,** 231-241. doi:10.1111/j.1752-7325.2009.00128.x.

Jones, A. A., Kornman, K. S., Newbold, D. A. & Manwell, M. A. (1994) Clinical and Microbiological Effects of Controlled-Release Locally Delivered Minocycline in Periodontitis. *Journal of Periodontology* **65,** 1058-1066.

Jordan, R. A., Lucaciu, A., Fotouhi, K., Markovic, L., Gaengler, P. & Zimmer, S. (2011) Pilot pathfinder survey of oral hygiene and periodontal conditions in the rural population of The Gambia (West Africa). *Int J Dent Hyg* **9,** 53-59. doi:10.1111/j.1601-5037.2009.00435.x.

Jordan, R. A., Lucaciu, A. & Zimmer, S. (2016) Influence of highly-active antiretroviral therapy on the subgingival biofilm in HIV-infected patients. *J Investig Clin Dent* **7,** 65-71.

Katagiri, S., Nitta, H., Nagasawa, T., Izumi, Y., Kanazawa, M., Matsuo, A., Chiba, H., Miyazaki, S., Miyauchi, T., Nakamura, N., Kanamura, N., Ando, Y., Hanada, N. & Inoue, S. (2010) High prevalence of periodontitis in non-elderly obese Japanese adults. *Obes Res Clin Pract* **4,** e247-342. doi:10.1016/j.orcp.2010.08.005.

Keller, J. J., Wu, C. S., Chen, Y. H. & Lin, H. C. (2013) Association between obstructive sleep apnoea and chronic periodontitis: a population-based study. *J Clin Periodontol* **40,** 111-117. doi:10.1111/jcpe.12036.

Kim, D. W., Park, J. C., Rim, T. T., Jung, U. W., Kim, C. S., Donos, N., Cha, I. H. & Choi, S. H. (2014a) Socioeconomic disparities of periodontitis in Koreans based on the KNHANES IV. *Oral Dis* **20,** 551-559. doi:10.1111/odi.12168.

Kim, E. J., Jin, B. H. & Bae, K. H. (2011) Periodontitis and obesity: a study of the Fourth Korean National Health and Nutrition Examination Survey. *J Periodontol* **82,** 533-542. doi:10.1902/jop.2010.100274.

Kim, H. S., Son, J. H., Yi, H. Y., Hong, H. K., Suh, H. J. & Bae, K. H. (2014b) Association between harmful alcohol use and periodontal status according to gender and smoking. *BMC Oral Health* **14,** 73. doi:10.1186/1472-6831-14-73.

Klein, R. S., Quart, A. M. & Small, C. B. (1991) Periodontal-Disease in Heterosexuals with Acquired-Immunodeficiency-Syndrome. *Journal of Periodontology* **62,** 535-540.

Kumar, S., Dagli, R. J., Dhanni, C. & Duraiswamy, P. (2009) Relationship of body mass index with periodontal health status of green marble. *Braz Oral Res* **23,** 365-369.

Lazaridou, E., Tsikrikoni, A., Fotiadou, C., Kyrmanidou, E., Vakirlis, E., Giannopoulou, C., Apalla, Z. & Ioannides, D. (2013) Association of chronic plaque psoriasis and severe periodontitis: a hospital based case-control study. *J Eur Acad Dermatol Venereol* **27,** 967-972. doi:10.1111/j.1468-3083.2012.04615.x.

Lee, H. J., Je, D. I., Won, S. J., Paik, D. I. & Bae, K. H. (2015) Association between vitamin D deficiency and periodontal status in current smokers. *Community Dent Oral Epidemiol* **43,** 471-478. doi:10.1111/cdoe.12173.

Lee, J. H., Shin, M. S., Kim, E. J., Ahn, Y. B. & Kim, H. D. (2017a) The association of dietary vitamin C intake with periodontitis among Korean adults: Results from KNHANES. *PLoS One* **12,** e0177074. doi:10.1371/journal.pone.0177074.

Lee, M., Choi, Y. H., Sagong, J., Yu, S., Kim, Y., Lee, D. & Kim, S. (2016) The interactive association of smoking and drinking levels with presence of periodontitis in South Korean adults. *BMC Oral Health* **16,** 80. doi:10.1186/s12903-016-0268-y.

Lee, W., Lim, S. S., Kim, B., Won, J. U., Roh, J. & Yoon, J. H. (2017b) Relationship between long working hours and periodontitis among the Korean. *Sci Rep* **7,** 7967.

Lewis, J. M., Morgan, M. V. & Wright, F. A. (1994) The validity of the CPITN scoring and presentation method for measuring periodontal conditions. *J Clin Periodontol* **21,** 1-6.

Liukkonen, J., Gursoy, U. K., Pussinen, P. J., Suominen, A. L. & Kononen, E. (2016) Salivary Concentrations of Interleukin (IL)-1beta, IL-17A, and IL-23 Vary in Relation to Periodontal Status. *J Periodontol* **87,** 1484-1491. doi:10.1902/jop.2016.160146.

Mattila, P. T., Niskanen, M. C., Vehkalahti, M. M., Nordblad, A. & Knuuttila, M. L. (2010) Prevalence and simultaneous occurrence of periodontitis and dental caries. *J Clin Periodontol* **37,** 962-967.

Melton, J. J., Redding, S. W., Kirkpatrick, W. R., Reasner, C. A., Ocampo, G. L., Venkatesh, A. & Mealey, B. L. (2010) Recovery of Candida dubliniensis and other Candida species from the oral cavity of subjects with periodontitis who had well-controlled and poorly controlled type 2 diabetes: a pilot study. *Spec Care Dentist* **30,** 230-234. doi:10.1111/j.1754-4505.2010.00159.x.

Merchant, A. T. (2012) Periodontitis and dental caries occur together. *J Evid Based Dent Pract* **12,** 18-19.

Miyazaki, H., Hanada, N., Andoh, M. I., Yamashita, Y., Saito, T., Sogame, A., Goto, K., Shirahama, R. & Takehara, T. (1989) Periodontal disease prevalence in different age groups in Japan as assessed according to the CPITN. *Community Dent Oral Epidemiol* **17,** 71-74.

Miyazaki, H., Yamashita, Y., Shirahama, R., Goto-Kimura, K., Shimada, N., Sogame, A. & Takehara, T. (1991) Periodontal condition of pregnant women assessed by CPITN. *J Clin Periodontol* **18,** 751-754.

Mohamed, H. G., Idris, S. B., Ahmed, M. F., Boe, O. E., Mustafa, K., Ibrahim, S. O. & Astrom, A. N. (2013) Association between oral health status and type 2 diabetes mellitus among Sudanese adults: a matched case-control study. *PLoS One* **8,** e82158. doi:10.1371/journal.pone.0082158.

Mombelli, A., Tonetti, M., Lehmann, B. & Lang, N. P. (1996) Topographic distribution of black-pigmenting anaerobes before and after periodontal treatment by local delivery of tetracycline. *Journal of Clinical Periodontology* **23,** 906-913.

Moosani, A., Sigal, M. J., Glogauer, M., Lawrence, H. P., Goldberg, M. & Tenenbaum, H. (2014) Evaluation of periodontal disease and oral inflammatory load in adults with. *Spec Care Dentist* **34,** 303-312.

Morita, I., Okamoto, Y., Yoshii, S., Nakagaki, H., Mizuno, K., Sheiham, A. & Sabbah, W. (2011) Five-year incidence of periodontal disease is related to body mass index. *J Dent Res* **90,** 199-202. doi:10.1177/0022034510382548.

Naorungroj, S., Schoenbach, V. J., Beck, J., Mosley, T. H., Gottesman, R. F., Alonso, A., Heiss, G. & Slade, G. D. (2013) Cross-sectional associations of oral health measures with cognitive function in late middle-aged adults: a community-based study. *J Am Dent Assoc* **144,** 1362-1371.

Natto, S., Baljoon, M. & Bergstrom, J. (2005) Tobacco smoking and periodontal health in a Saudi Arabian population. *J Periodontol* **76,** 1919-1926. doi:10.1902/jop.2005.76.11.1919.

Nesse, W., Dijkstra, P. U., Abbas, F., Spijkervet, F. K., Stijger, A., Tromp, J. A., van Dijk, J. L. & Vissink, A. (2010) Increased prevalence of cardiovascular and autoimmune diseases in periodontitis patients: a cross-sectional study. *J Periodontol* **81,** 1622-1628. doi:10.1902/jop.2010.100058.

Norderyd, O., Henriksen, B. M. & Jansson, H. (2012) Periodontal disease in Norwegian old-age pensioners. *Gerodontology* **29,** 4-8.

Norderyd, O., Koch, G., Papias, A., Kohler, A. A., Helkimo, A. N., Brahm, C. O., Lindmark, U., Lindfors, N., Mattsson, A., Rolander, B., Ullbro, C., Gerdin, E. W. & Frisk, F. (2015) Oral health of individuals aged 3-80 years in Jonkoping, Sweden during 40 years (1973-2013). II. Review of clinical and radiographic findings. *Swed Dent J* **39,** 69-86.

Okamoto, Y., Tsuboi, S., Suzuki, S., Nakagaki, H., Ogura, Y., Maeda, K. & Tokudome, S. (2006) Effects of smoking and drinking habits on the incidence of periodontal disease. *J Periodontal Res* **41,** 560-566.

Ozcaka, O., Becerik, S., Bicakci, N. & Kiyak, A. H. (2014) Periodontal disease and systemic diseases in an older population. *Arch Gerontol Geriatr* **59,** 474-479.

Park, J. A., Lee, J. H., Lee, H. J., Jin, B. H. & Bae, K. H. (2017) Association of Some Vitamins and Minerals with Periodontitis in a Nationally Representative Sample of Korean Young Adults. *Biol Trace Elem Res* **178,** 171-179. doi:10.1007/s12011-016-0914-x.

Park, J. B., Han, K., Park, Y. G. & Ko, Y. (2014) Association between alcohol consumption and periodontal disease: the 2008 to 2010 Korea National Health and Nutrition Examination Survey. *J Periodontol* **85,** 1521-1528. doi:10.1902/jop.2014.130782.

Persson, R., Svendsen, J. & Daubert, K. (1989) A longitudinal evaluation of periodontal therapy using the CPITN index. *J Clin Periodontol* **16,** 569-574.

Pilot, T., Lu, Z. Y., Lin, Z. Q., Yen, W. P. & Cao, G. R. (1989) Periodontal conditions in 35-44-year-old factory workers in Shanghai. *Community Dent Oral Epidemiol* **17,** 216.

Preshaw, P. M., de Silva, N., McCracken, G. I., Fernando, D. J., Dalton, C. F., Steen, N. D. & Heasman, P. A. (2010) Compromised periodontal status in an urban Sri Lankan population with type 2 diabetes. *J Clin Periodontol* **37,** 165-171. doi:10.1111/j.1600-051X.2009.01519.x.

Psoter, W. J., Ge, Y., Russell, S. L., Chen, Z., Katz, R. V., Jean-Charles, G. & Li, Y. (2011) PCR detection of Streptococcus mutans and Aggregatibacter actinomycetemcomitans in dental plaque samples from Haitian adolescents. *Clin Oral Investig* **15,** 461-469. doi:10.1007/s00784-010-0413-y.

Pumerantz, A. S., Bissett, S. M., Dong, F., Ochoa, C., Wassall, R. R., Davila, H., Barbee, M., Nguyen, J., Vila, P. & Preshaw, P. M. (2017) Standardized screening for periodontitis as an integral part of multidisciplinary management of adults with type 2 diabetes: an observational cross-sectional study of cohorts in the USA and UK. *BMJ Open Diabetes Res Care* **5,** e000413. doi:10.1136/bmjdrc-2017-000413.

Ramseier, C. A., Mirra, D., Schutz, C., Sculean, A., Lang, N. P., Walter, C. & Salvi, G. E. (2015) Bleeding on probing as it relates to smoking status in patients enrolled in supportive periodontal therapy for at least 5 years. *J Clin Periodontol* **42,** 150-159. doi:10.1111/jcpe.12344.

Romandini, M., Gioco, G., Perfetti, G., Deli, G., Staderini, E. & Lafori, A. (2017) The association between periodontitis and sleep duration. *J Clin Periodontol* **44,** 490-501. doi:10.1111/jcpe.12713.

Sabbah, W., Sheiham, A. & Bernabe, E. (2010) Income inequality and periodontal diseases in rich countries: an ecological. *Int Dent J* **60,** 370-374.

Sanchez-Siles, M., Rosa-Salazar, V., Camacho-Alonso, F., Salazar-Sanchez, N. & Cozar-Hidalgo, J. (2013) Association between periodontal disease and venous thromboembolic disease. *Quintessence Int* **44,** 567-573. doi:10.3290/j.qi.a29749.

Saxlin, T., Ylostalo, P., Suominen-Taipale, L., Aromaa, A. & Knuuttila, M. (2010) Overweight and obesity weakly predict the development of periodontal infection. *J Clin Periodontol* **37,** 1059-1067.

Saxlin, T., Ylostalo, P., Suominen-Taipale, L., Mannisto, S. & Knuuttila, M. (2011) Association between periodontal infection and obesity: results of the Health 2000. *J Clin Periodontol* **38,** 236-242.

Shin, B. M., Ryu, J. I., Sheiham, A., Do, L. G. & Jung, S. H. (2015) Which life course model better explains the association between socioeconomic position and periodontal health? *J Clin Periodontol* **42,** 213-220. doi:10.1111/jcpe.12360.

Sicilia, A., Cobo, J., Sanz, M., Noguerol, B., Ainamo, J., Bascones, A. & Lopez Arranz, J. S. (1990) Periodontal treatment needs in the young population in Oviedo, Spain. *Community Dent Oral Epidemiol* **18,** 223-224.

Sim, H. Y., Kim, H. S., Jung, D. U., Lee, H., Lee, J. W., Han, K. & Yun, K. I. (2017) Association between orthodontic treatment and periodontal diseases: Results from a national survey. *Angle Orthod* **87,** 651-657. doi:10.2319/030317-162.1.

Sjostrom, L., Laurell, L., Hugoson, A. & Hakansson, J. P. (1989) Periodontal conditions in adults with rheumatoid arthritis. *Community Dent Oral Epidemiol* **17,** 234-236.

Tanaka, K., Miyake, Y., Hanioka, T. & Arakawa, M. (2013) Active and passive smoking and prevalence of periodontal disease in young Japanese women. *J Periodontal Res* **48,** 600-605. doi:10.1111/jre.12044.

Tellapragada, C., Eshwara, V. K., Acharya, S., Bhat, P., Kamath, A., Vishwanath, S. & Mukhopadhyay, C. (2014) Prevalence of Clinical Periodontitis and Putative Periodontal Pathogens among South Indian Pregnant Women. *Int J Microbiol* **2014,** 420149. doi:10.1155/2014/420149.

Tervonen, T., Knuuttila, M. & Nieminen, P. (1991) Risk factors associated with abundant dental caries and periodontal pocketing. *Community Dent Oral Epidemiol* **19,** 82-87.

Thomson, W. M., Broadbent, J. M., Poulton, R. & Beck, J. D. (2006) Changes in periodontal disease experience from 26 to 32 years of age in a birth cohort. *J Periodontol* **77,** 947-954. doi:10.1902/jop.2006.050319.

Thorstensson, H., Dahlen, G. & Hugoson, A. (1995) Some Suspected Periodontopathogens and Serum Antibody-Response in Adult Long-Duration Insulin-Dependent Diabetics. *Journal of Clinical Periodontology* **22,** 449-458.

Timonen, P., Niskanen, M., Suominen-Taipale, L., Jula, A., Knuuttila, M. & Ylostalo, P. (2010) Metabolic syndrome, periodontal infection, and dental caries. *J Dent Res* **89,** 1068-1073. doi:10.1177/0022034510376542.

Timonen, P., Suominen-Taipale, L., Jula, A., Niskanen, M., Knuuttila, M. & Ylostalo, P. (2011) Insulin sensitivity and periodontal infection in a non-diabetic, non-smoking adult population. *J Clin Periodontol* **38,** 17-24. doi:10.1111/j.1600-051X.2010.01642.x.

Torrungruang, K., Bandhaya, P., Likittanasombat, K. & Grittayaphong, C. (2009) Relationship between the presence of certain bacterial pathogens and periodontal status of urban Thai adults. *J Periodontol* **80,** 122-129. doi:10.1902/jop.2009.080248.

Toygar, H. U., Seydaoglu, G., Kurklu, S., Guzeldemir, E. & Arpak, N. (2007) Periodontal health and adverse pregnancy outcome in 3,576 Turkish women. *J Periodontol* **78,** 2081-2094. doi:10.1902/jop.2007.070092.

Trombelli, L., Rizzi, A., Simonelli, A., Scapoli, C., Carrieri, A. & Farina, R. (2010) Age-related treatment response following non-surgical periodontal therapy. *J Clin Periodontol* **37,** 346-352. doi:10.1111/j.1600-051X.2010.01541.x.

Vano, M., Gennai, S., Karapetsa, D., Miceli, M., Giuca, M. R., Gabriele, M. & Graziani, F. (2015) The influence of educational level and oral hygiene behaviours on DMFT index and CPITN index in an adult Italian population: an epidemiological study. *Int J Dent Hyg* **13,** 151-157. doi:10.1111/idh.12098.

Vered, Y., Livny, A., Zini, A. & Sgan-Cohen, H. D. (2008) Periodontal health status and smoking among young adults. *J Clin Periodontol* **35,** 768-772. doi:10.1111/j.1600-051X.2008.01294.x.

Wara-aswapati, N., Pitiphat, W., Chanchaimongkon, L., Taweechaisupapong, S., Boch, J. A. & Ishikawa, I. (2009) Red bacterial complex is associated with the severity of chronic periodontitis in a Thai population. *Oral Dis* **15,** 354-359. doi:10.1111/j.1601-0825.2009.01562.x.

Ylostalo, P., Suominen-Taipale, L., Reunanen, A. & Knuuttila, M. (2008) Association between body weight and periodontal infection. *J Clin Periodontol* **35,** 297-304. doi:10.1111/j.1600-051X.2008.01203.x.

Zhang, Q., Li, Z., Wang, C., Shen, T., Yang, Y., Chotivichien, S. & Wang, L. (2014) Prevalence and predictors for periodontitis among adults in China, 2010. *Glob Health Action* **7,** 24503. doi:10.3402/gha.v7.24503.

Ziebolz, D., Pabel, S. O., Lange, K., Krohn-Grimberghe, B., Hornecker, E. & Mausberg, R. F. (2011) Clinical periodontal and microbiologic parameters in patients with rheumatoid arthritis. *J Periodontol* **82,** 1424-1432. doi:10.1902/jop.2011.100481.

*Supplementary Appendix : ICD*

Choi, J. K., Kim, Y. T., Kweon, H. I., Park, E. C., Choi, S. H. & Lee, J. H. (2017) Effect of periodontitis on the development of osteoporosis: results from a nationwide population-based cohort study (2003-2013). *BMC Womens Health* **17,** 77. doi:10.1186/s12905-017-0440-9.

*Supplementary Appendix : PD+CAL*

Aemaimanan, P., Sattayasai, N., Wara-aswapati, N., Pitiphat, W., Suwannarong, W., Prajaneh, S. & Taweechaisupapong, S. (2009) Alanine aminopeptidase and dipeptidyl peptidase IV in saliva of chronic periodontitis patients. *J Periodontol* **80,** 1809-1814. doi:10.1902/jop.2009.090233.

Ahn, Y. B., Shin, M. S., Han, D. H., Sukhbaatar, M., Kim, M. S., Shin, H. S. & Kim, H. D. (2016) Periodontitis is associated with the risk of subclinical atherosclerosis and peripheral arterial disease in Korean adults. *Atherosclerosis* **251,** 311-318. doi:10.1016/j.atherosclerosis.2016.07.898.

Aimetti, M., Perotto, S., Castiglione, A., Mariani, G. M., Ferrarotti, F. & Romano, F. (2015) Prevalence of periodontitis in an adult population from an urban area in North Italy: findings from a cross-sectional population-based epidemiological survey. *J Clin Periodontol* **42,** 622-631. doi:10.1111/jcpe.12420.

Akinkugbe, A. A., Slade, G. D., Barritt, A. S., Cole, S. R., Offenbacher, S., Petersmann, A., Kocher, T., Lerch, M. M., Mayerle, J., Volzke, H., Heiss, G. & Holtfreter, B. (2017) Periodontitis and Non-alcoholic Fatty Liver Disease, a population-based cohort investigation in the Study of Health in Pomerania. *J Clin Periodontol* **44,** 1077-1087. doi:10.1111/jcpe.12800.

Al-Zahrani, M. S. (2006) Increased intake of dairy products is related to lower periodontitis prevalence. *J Periodontol* **77,** 289-294. doi:10.1902/jop.2006.050082.

Al-Zahrani, M. S., Borawski, E. A. & Bissada, N. F. (2005) Periodontitis and three health-enhancing behaviors: maintaining normal weight, engaging in recommended level of exercise, and consuming a high-quality diet. *J Periodontol* **76,** 1362-1366. doi:10.1902/jop.2005.76.8.1362.

Antoniazzi, R. P., Zanatta, F. B., Rosing, C. K. & Feldens, C. A. (2016) Association Among Periodontitis and the Use of Crack Cocaine and Other Illicit Drugs. *J Periodontol* **87,** 1396-1405. doi:10.1902/jop.2016.150732.

Arteaga-Guerra, J. J., Ceron-Souza, V. & Mafla, A. C. (2010) Dynamic among periodontal disease, stress, and adverse pregnancy outcomes. *Rev Salud Publica (Bogota)* **12,** 276-286.

Barbosa, F. C., Irino, K., Carbonell, G. V. & Mayer, M. P. (2006) Characterization of Serratia marcescens isolates from subgingival biofilm, extraoral infections and environment by prodigiosin production, serotyping, and genotyping. *Oral Microbiol Immunol* **21,** 53-60. doi:10.1111/j.1399-302X.2005.00254.x.

Bawadi, H. A., Khader, Y. S., Haroun, T. F., Al-Omari, M. & Tayyem, R. F. (2011) The association between periodontal disease, physical activity and healthy diet among adults in Jordan. *J Periodontal Res* **46,** 74-81. doi:10.1111/j.1600-0765.2010.01314.x.

Beck, J. D., Koch, G. G., Zambon, J. J., Genco, R. J. & Tudor, G. E. (1992) Evaluation of Oral Bacteria as Risk Indicators for Periodontitis in Older Adults. *Journal of Periodontology* **63,** 93-99.

Bilichodmath, S., Mangalekar, S. B., Sharma, D. C., Prabhakar, A. K., Reddy, S. B., Kalburgi, N. B., Patil, S. R. & Bhat, K. (2009) Herpesviruses in chronic and aggressive periodontitis patients in an Indian population. *J Oral Sci* **51,** 79-86.

Borrell, L. N., Beck, J. D. & Heiss, G. (2006a) Socioeconomic disadvantage and periodontal disease: the Dental Atherosclerosis Risk in Communities study. *Am J Public Health* **96,** 332-339. doi:10.2105/ajph.2004.055277.

Borrell, L. N., Burt, B. A., Neighbors, H. W. & Taylor, G. W. (2008) Social factors and periodontitis in an older population. *Am J Public Health* **98,** S95-101.

Borrell, L. N., Burt, B. A. & Taylor, G. W. (2005) Prevalence and trends in periodontitis in the USA: the [corrected] NHANES, 1988 to 2000. *J Dent Res* **84,** 924-930. doi:10.1177/154405910508401010.

Borrell, L. N., Burt, B. A., Warren, R. C. & Neighbors, H. W. (2006b) The role of individual and neighborhood social factors on periodontitis: the third National Health and Nutrition Examination Survey. *J Periodontol* **77,** 444-453. doi:10.1902/jop.2006.050158.

Borrell, L. N. & Crawford, N. D. (2008) Social disparities in periodontitis among United States adults 1999-2004. *Community Dent Oral Epidemiol* **36,** 383-391.

Borrell, L. N., Kunzel, C., Lamster, I. & Lalla, E. (2007) Diabetes in the dental office: using NHANES III to estimate the probability of undiagnosed disease. *J Periodontal Res* **42,** 559-565. doi:10.1111/j.1600-0765.2007.00983.x.

Boutin, S., Hagenfeld, D., Zimmermann, H., El Sayed, N., Hopker, T., Greiser, H. K., Becher, H., Kim, T. S. & Dalpke, A. H. (2017) Clustering of Subgingival Microbiota Reveals Microbial Disease Ecotypes Associated with Clinical Stages of Periodontitis in a Cross-Sectional Study. *Front Microbiol* **8,** 340. doi:10.3389/fmicb.2017.00340.

Brennan, D. S., Spencer, A. J. & Roberts-Thomson, K. F. (2007) Periodontal disease among 45-54 year olds in Adelaide, South Australia. *Aust Dent J* **52,** 55-60.

Chapple, I. L., Milward, M. R. & Dietrich, T. (2007) The prevalence of inflammatory periodontitis is negatively associated with serum antioxidant concentrations. *J Nutr* **137,** 657-664.

Chen, H., Liu, Y., Zhang, M., Wang, G., Qi, Z., Bridgewater, L., Zhao, L., Tang, Z. & Pang, X. (2015) A Filifactor alocis-centered co-occurrence group associates with periodontitis across different oral habitats. *Sci Rep* **5,** 9053. doi:10.1038/srep09053.

Chen, Y. W., Nagasawa, T., Wara-Aswapati, N., Ushida, Y., Wang, D., Takeuchi, Y., Kobayashi, H., Umeda, M., Inoue, Y., Iwai, T., Ishikawa, I. & Izumi, Y. (2009) Association between periodontitis and anti-cardiolipin antibodies in Buerger disease. *J Clin Periodontol* **36,** 830-835. doi:10.1111/j.1600-051X.2009.01467.x.

Contreras, A., Herrera, J. A., Soto, J. E., Arce, R. M., Jaramillo, A. & Botero, J. E. (2006) Periodontitis is associated with preeclampsia in pregnant women. *J Periodontol* **77,** 182-188. doi:10.1902/jop.2006.050020.

Cota, L. O., Guimaraes, A. N., Costa, J. E., Lorentz, T. C. & Costa, F. O. (2006) Association between maternal periodontitis and an increased risk of preeclampsia. *J Periodontol* **77,** 2063-2069. doi:10.1902/jop.2006.060061.

D'Aiuto, F., Sabbah, W., Netuveli, G., Donos, N., Hingorani, A. D., Deanfield, J. & Tsakos, G. (2008) Association of the metabolic syndrome with severe periodontitis in a large U.S. population-based survey. *J Clin Endocrinol Metab* **93,** 3989-3994. doi:10.1210/jc.2007-2522.

Danser, M. M., Timmerman, M. F., vanWinkelhoff, A. J. & vanderVelden, U. (1996) The effect of periodontal treatment on periodontal bacteria on the oral mucous membranes. *Journal of Periodontology* **67,** 478-485.

de Macedo, T. C., Costa Mda, C., Gomes-Filho, I. S., Vianna, M. I. & Santos, C. T. (2006) Factors related to periodontal disease in a rural population. *Braz Oral Res* **20,** 257-262.

Diouf, M., Basse, A., Ndiaye, M., Cisse, D., Lo, C. M. & Faye, D. (2015) Stroke and periodontal disease in Senegal: case-control study. *Public Health* **129,** 1669-1673. doi:10.1016/j.puhe.2015.02.033.

Eke, P. I., Wei, L., Thornton-Evans, G. O., Borrell, L. N., Borgnakke, W. S., Dye, B. & Genco, R. J. (2016) Risk Indicators for Periodontitis in US Adults: NHANES 2009 to 2012. *J Periodontol* **87,** 1174-1185. doi:10.1902/jop.2016.160013.

El Attar, M. M., Zaghloup, M. Z. & Elmenoufr, H. S. (2010) Role of periodontitis in hospital-acquired pneumonia. *East Mediterr Health J* **16,** 563-569.

Fitzsimmons, T. R., Sanders, A. E., Slade, G. D. & Bartold, P. M. (2009) Biomarkers of periodontal inflammation in the Australian adult population. *Aust Dent J* **54,** 115-122. doi:10.1111/j.1834-7819.2009.01103.x.

Gamsiz-Isik, H., Kiyan, E., Bingol, Z., Baser, U., Ademoglu, E. & Yalcin, F. (2017) Does Obstructive Sleep Apnea Increase the Risk for Periodontal Disease? A Case-Control Study. *J Periodontol* **88,** 443-449. doi:10.1902/jop.2016.160365.

Garcia, D., Tarima, S. & Okunseri, C. (2015) Periodontitis and glycemic control in diabetes: NHANES 2009 to 2012. *J Periodontol* **86,** 499-506. doi:10.1902/jop.2014.140364.

Goulart, A. C., Armani, F., Arap, A. M., Nejm, T., Andrade, J. B., Bufarah, H. B. & Dezen, D. H. S. (2017) Relationship between periodontal disease and cardiovascular risk factors among young and middle-aged Brazilians. Cross-sectional study. *Sao Paulo Med J* **135,** 226-233. doi:10.1590/1516-3180.2016.0357300117.

Grubbs, V., Vittinghoff, E., Beck, J. D., Kshirsagar, A. V., Wang, W., Griswold, M. E., Powe, N. R., Correa, A. & Young, B. (2015) Association Between Periodontal Disease and Kidney Function Decline in African Americans: The Jackson Heart Study. *J Periodontol* **86,** 1126-1132. doi:10.1902/jop.2015.150195.

Gunaratnam, K., Taylor, B., Curtis, B. & Cistulli, P. (2009) Obstructive sleep apnoea and periodontitis: a novel association? *Sleep Breath* **13,** 233-239. doi:10.1007/s11325-008-0244-0.

Habashneh, R. A., Khader, Y. S., Alhumouz, M. K., Jadallah, K. & Ajlouni, Y. (2012) The association between inflammatory bowel disease and periodontitis among Jordanians: a case-control study. *J Periodontal Res* **47,** 293-298. doi:10.1111/j.1600-0765.2011.01431.x.

Heaton, B., Gordon, N. B., Garcia, R. I., Rosenberg, L., Rich, S., Fox, M. P. & Cozier, Y. C. (2017) A Clinical Validation of Self-Reported Periodontitis Among Participants in the Black Women's Health Study. *J Periodontol* **88,** 582-592. doi:10.1902/jop.2017.160678.

Hernandez-Vigueras, S., Martinez-Garriga, B., Sanchez, M. C., Sanz, M., Estrugo-Devesa, A., Vinuesa, T., Lopez-Lopez, J. & Vinas, M. (2016) Oral Microbiota, Periodontal Status, and Osteoporosis in Postmenopausal Females. *J Periodontol* **87,** 124-133. doi:10.1902/jop.2015.150365.

Herrera, D., Contreras, A., Gamonal, J., Oteo, A., Jaramillo, A., Silva, N., Sanz, M., Botero, J. E. & Leon, R. (2008) Subgingival microbial profiles in chronic periodontitis patients from Chile, Colombia and Spain. *J Clin Periodontol* **35,** 106-113. doi:10.1111/j.1600-051X.2007.01170.x.

Hirotomi, T., Yoshihara, A., Ogawa, H., Ito, K., Igarashi, A. & Miyazaki, H. (2006) A preliminary study on the relationship between stimulated saliva and periodontal conditions in community-dwelling elderly people. *J Dent* **34,** 692-698. doi:10.1016/j.jdent.2006.01.001.

Ho, Y. P., Lin, Y. C., Yang, Y. H., Ho, K. Y., Wu, Y. M. & Tsai, C. C. (2008) Cyclooxygenase-2 Gene-765 single nucleotide polymorphism as a protective factor against periodontitis in Taiwanese. *J Clin Periodontol* **35,** 1-8. doi:10.1111/j.1600-051X.2007.01167.x.

Holde, G. E., Oscarson, N., Trovik, T. A., Tillberg, A. & Jonsson, B. (2017) Periodontitis Prevalence and Severity in Adults: A Cross-Sectional Study in Norwegian Circumpolar Communities. *J Periodontol* **88,** 1012-1022. doi:10.1902/jop.2017.170164.

Holtfreter, B., Albandar, J. M., Dietrich, T., Dye, B. A., Eaton, K. A., Eke, P. I., Papapanou, P. N. & Kocher, T. (2015) Standards for reporting chronic periodontitis prevalence and severity in epidemiologic studies: Proposed standards from the Joint EU/USA Periodontal Epidemiology Working Group. *J Clin Periodontol* **42,** 407-412. doi:10.1111/jcpe.12392.

Holtfreter, B., Schwahn, C., Biffar, R. & Kocher, T. (2009) Epidemiology of periodontal diseases in the Study of Health in Pomerania. *J Clin Periodontol* **36,** 114-123. doi:10.1111/j.1600-051X.2008.01361.x.

Holzhausen, M., Cortelli, J. R., da Silva, V. A., Franco, G. C., Cortelli, S. C. & Vergnolle, N. (2010) Protease-activated receptor-2 (PAR(2)) in human periodontitis. *J Dent Res* **89,** 948-953. doi:10.1177/0022034510373765.

Horewicz, V. V., Feres, M., Rapp, G. E., Yasuda, V. & Cury, P. R. (2010) Human papillomavirus-16 prevalence in gingival tissue and its association with periodontal destruction: a case-control study. *J Periodontol* **81,** 562-568. doi:10.1902/jop.2009.090571.

Houshmand, M., Holtfreter, B., Berg, M. H., Schwahn, C., Meisel, P., Biffar, R., Kindler, S. & Kocher, T. (2012) Refining definitions of periodontal disease and caries for prediction models of incident tooth loss. *J Clin Periodontol* **39,** 635-644. doi:10.1111/j.1600-051X.2012.01892.x.

Ide, M., Harris, M., Stevens, A., Sussams, R., Hopkins, V., Culliford, D., Fuller, J., Ibbett, P., Raybould, R., Thomas, R., Puenter, U., Teeling, J., Perry, V. H. & Holmes, C. (2016) Periodontitis and Cognitive Decline in Alzheimer's Disease. *PLoS One* **11,** e0151081. doi:10.1371/journal.pone.0151081.

Joshi, V. M., Bhat, K. G., Kugaji, M. S. & Shirahatti, R. (2017) Characterization and serotype distribution of Aggregatibacter actinomycetemcomitans: Relationship of serotypes to herpesvirus and periodontal status in Indian subjects. *Microb Pathog* **110,** 189-195. doi:10.1016/j.micpath.2017.06.041.

Kapellas, K., Maple-Brown, L. J., Jamieson, L. M., Do, L. G., O'Dea, K., Brown, A., Cai, T. Y., Anstey, N. M., Sullivan, D. R., Wang, H., Celermajer, D. S., Slade, G. D. & Skilton, M. R. (2014a) Effect of periodontal therapy on arterial structure and function among aboriginal australians: a randomized, controlled trial. *Hypertension* **64,** 702-708. doi:10.1161/hypertensionaha.114.03359.

Kapellas, K., Skilton, M. R., Maple-Brown, L. J., Do, L. G., Bartold, P. M., O'Dea, K., Brown, A., Celermajer, D. S. & Jamieson, L. M. (2014b) Periodontal disease and dental caries among Indigenous Australians living in the Northern Territory, Australia. *Aust Dent J* **59,** 93-99. doi:10.1111/adj.12135.

Kassab, P., Colombier, M. L., Kaminski, M., Lelong, N., Sixou, M. & Nabet, C. (2011) Impact of periodontitis definition in epidemiological research. Results from the EPIPAP study in postpartum women. *Eur J Oral Sci* **119,** 156-162. doi:10.1111/j.1600-0722.2011.00816.x.

Kavoussi, S. K., West, B. T., Taylor, G. W. & Lebovic, D. I. (2009) Periodontal disease and endometriosis: analysis of the National Health and Nutrition Examination Survey. *Fertil Steril* **91,** 335-342. doi:10.1016/j.fertnstert.2007.12.075.

Khader, Y. S., Bawadi, H. A., Haroun, T. F., Alomari, M. & Tayyem, R. F. (2009) The association between periodontal disease and obesity among adults in Jordan. *J Clin Periodontol* **36,** 18-24. doi:10.1111/j.1600-051X.2008.01345.x.

Khantisopon, N., Louthrenoo, W., Kasitanon, N., Sivasomboon, C., Wangkaew, S., Sang-In, S., Jotikasthira, N. & Bandhaya, P. (2014) Periodontal disease in Thai patients with rheumatoid arthritis. *Int J Rheum Dis* **17,** 511-518. doi:10.1111/1756-185x.12315.

Kim, J. K., Baker, L. A., Seirawan, H. & Crimmins, E. M. (2012) Prevalence of oral health problems in U.S. adults, NHANES 1999-2004: exploring differences by age, education, and race/ethnicity. *Spec Care Dentist* **32,** 234-241. doi:10.1111/j.1754-4505.2012.00280.x.

Kotsakis, G. A., Thai, A., Ioannou, A. L., Demmer, R. T. & Michalowicz, B. S. (2015) Association between low-dose aspirin and periodontal disease: results from the continuous national health and nutrition examination survey (NHANES) 2011-2012. *J Clin Periodontol* **42,** 333-341. doi:10.1111/jcpe.12380.

Lee, D. H., Jacobs, D. R. & Kocher, T. (2008) Associations of serum concentrations of persistent organic pollutants with the prevalence of periodontal disease and subpopulations of white blood cells. *Environ Health Perspect* **116,** 1558-1562. doi:10.1289/ehp.11425.

Leira, Y., Ameijeira, P., Dominguez, C., Leira, R. & Blanco, J. (2017) The role of leptin as a biomarker in the relationship between periodontitis and chronic migraine. *J Clin Periodontol* **44,** 1208-1214. doi:10.1111/jcpe.12819.

Linden, G., Patterson, C., Evans, A. & Kee, F. (2007) Obesity and periodontitis in 60-70-year-old men. *J Clin Periodontol* **34,** 461-466. doi:10.1111/j.1600-051X.2007.01075.x.

Linden, G. J., Linden, K., Yarnell, J., Evans, A., Kee, F. & Patterson, C. C. (2012) All-cause mortality and periodontitis in 60-70-year-old men: a prospective cohort study. *J Clin Periodontol* **39,** 940-946. doi:10.1111/j.1600-051X.2012.01923.x.

Linden, G. J., McClean, K., Young, I., Evans, A. & Kee, F. (2008) Persistently raised C-reactive protein levels are associated with advanced periodontal disease. *J Clin Periodontol* **35,** 741-747. doi:10.1111/j.1600-051X.2008.01288.x.

Linden, G. J., McClean, K. M., Woodside, J. V., Patterson, C. C., Evans, A., Young, I. S. & Kee, F. (2009) Antioxidants and periodontitis in 60-70-year-old men. *J Clin Periodontol* **36,** 843-849. doi:10.1111/j.1600-051X.2009.01468.x.

Machida, T., Tomofuji, T., Ekuni, D., Azuma, T., Takeuchi, N., Maruyama, T., Mizutani, S., Kataoka, K., Kawabata, Y. & Morita, M. (2014) Severe periodontitis is inversely associated with coffee consumption in the maintenance phase of periodontal treatment. *Nutrients* **6,** 4476-4490. doi:10.3390/nu6104476.

Martinez-Pabon, M. C., Martinez-Gaviria, A., Isaza-Guzman, D. M., Muskus-Lopez, C. E. & Tobon-Arroyave, S. I. (2010) Confounding and interaction effect of Treponema denticola salivary carriage in chronic periodontitis. *Oral Dis* **16,** 278-285. doi:10.1111/j.1601-0825.2009.01639.x.

Marulanda, A. M., Coral, D., Sabogal, D. & Serrano, C. (2014) Periodontal conditions of Colombian university students aged 16 to 35. *Braz Oral Res* **28**.

Meqa, K., Dragidella, F., Disha, M. & Sllamniku-Dalipi, Z. (2017) The Association between Periodontal Disease and Preterm Low Birthweight in Kosovo. *Acta Stomatol Croat* **51,** 33-40. doi:10.15644/asc51/1/4.

Minagawa, K., Iwasaki, M., Ogawa, H., Yoshihara, A. & Miyazaki, H. (2015) Relationship between metabolic syndrome and periodontitis in 80-year-old Japanese subjects. *J Periodontal Res* **50,** 173-179. doi:10.1111/jre.12190.

Moscicki, A. B., Yao, T. J., Ryder, M. I., Russell, J. S., Dominy, S. S., Patel, K., McKenna, M., Van Dyke, R. B., Seage, G. R., 3rd & Hazra, R. (2016) The Burden of Oral Disease among Perinatally HIV-Infected and HIV-Exposed Uninfected Youth. *PLoS One* **11,** e0156459. doi:10.1371/journal.pone.0156459.

Munoz-Torres, F. J., Jimenez, M. C., Rivas-Tumanyan, S. & Joshipura, K. J. (2014) Associations between measures of central adiposity and periodontitis among older adults. *Community Dent Oral Epidemiol* **42,** 170-177. doi:10.1111/cdoe.12069.

Musskopf, M. L., Daudt, L. D., Weidlich, P., Gerchman, F., Gross, J. L. & Oppermann, R. V. (2017) Metabolic syndrome as a risk indicator for periodontal disease and tooth loss. *Clin Oral Investig* **21,** 675-683. doi:10.1007/s00784-016-1935-8.

Naorungroj, S., Slade, G. D., Divaris, K., Heiss, G., Offenbacher, S. & Beck, J. D. (2017) Racial differences in periodontal disease and 10-year self-reported tooth loss among late middle-aged and older adults: the dental ARIC study. *J Public Health Dent* **77,** 372-382. doi:10.1111/jphd.12226.

Naqvi, A. Z., Buettner, C., Phillips, R. S., Davis, R. B. & Mukamal, K. J. (2010) n-3 fatty acids and periodontitis in US adults. *J Am Diet Assoc* **110,** 1669-1675. doi:10.1016/j.jada.2010.08.009.

Nielsen, S. J., Trak-Fellermeier, M. A., Joshipura, K. & Dye, B. A. (2016) Dietary Fiber Intake Is Inversely Associated with Periodontal Disease among US Adults. *J Nutr* **146,** 2530-2536. doi:10.3945/jn.116.237065.

Novak, M. J., Potter, R. M., Blodgett, J. & Ebersole, J. L. (2008) Periodontal disease in Hispanic Americans with type 2 diabetes. *J Periodontol* **79,** 629-636. doi:10.1902/jop.2008.070442.

Oliveira, R. R., Fermiano, D., Feres, M., Figueiredo, L. C., Teles, F. R., Soares, G. M. & Faveri, M. (2016) Levels of Candidate Periodontal Pathogens in Subgingival Biofilm. *J Dent Res* **95,** 711-718. doi:10.1177/0022034516634619.

Petit, M. D. A., Vansteenbergen, T. J. M., Timmerman, M. F., Degraaff, J. & Vandervelden, U. (1994) Prevalence of Periodontitis and Suspected Periodontal Pathogens in Families of Adult Periodontitis Patients. *Journal of Clinical Periodontology* **21,** 76-85.

Pischon, N., Hoedke, D., Kurth, S., Lee, P., Dommisch, H., Steinbrecher, A., Pischon, T., Burmester, G. R., Buttgereit, F., Detert, J. & Riemekasten, G. (2016) Increased Periodontal Attachment Loss in Patients With Systemic Sclerosis. *J Periodontol* **87,** 763-771. doi:10.1902/jop.2016.150475.

Piscoya, M. D., Ximenes, R. A., Silva, G. M., Jamelli, S. R. & Coutinho, S. B. (2012) Periodontitis-associated risk factors in pregnant women. *Clinics (Sao Paulo)* **67,** 27-33.

Puig-Silla, M., Montiel-Company, J. M., Dasi-Fernandez, F. & Almerich-Silla, J. M. (2017) Prevalence of periodontal pathogens as predictor of the evolution of periodontal status. *Odontology* **105,** 467-476. doi:10.1007/s10266-016-0286-x.

Rivas-Tumanyan, S., Campos, M., Zevallos, J. C. & Joshipura, K. J. (2013) Periodontal disease, hypertension, and blood pressure among older adults in Puerto Rico. *J Periodontol* **84,** 203-211. doi:10.1902/jop.2012.110748.

Riviere, G. R., Smith, K. S., Carranza, N., Tzagaroulaki, E., Kay, S. L. & Dock, M. (1995) Subgingival Distribution of Treponema-Denticola, Treponema-Socranskii, and Pathogen-Related Oral Spirochetes - Prevalence and Relationship to Periodontal Status of Sampled Sites. *Journal of Periodontology* **66,** 829-837.

Ryder, M. I., Yao, T. J., Russell, J. S., Moscicki, A. B. & Shiboski, C. H. (2017) Prevalence of periodontal diseases in a multicenter cohort of perinatally HIV-infected and HIV-exposed and uninfected youth. *J Clin Periodontol* **44,** 2-12. doi:10.1111/jcpe.12646.

Sabbah, W., Tsakos, G., Sheiham, A. & Watt, R. G. (2009) The effects of income and education on ethnic differences in oral health: a study in US adults. *J Epidemiol Community Health* **63,** 516-520. doi:10.1136/jech.2008.082313.

Sanders, A. & Slade, G. (2013) State cigarette excise tax, secondhand smoke exposure, and periodontitis in US nonsmokers. *Am J Public Health* **103,** 740-746. doi:10.2105/ajph.2011.300579.

Sanders, A. E., Essick, G. K., Beck, J. D., Cai, J., Beaver, S., Finlayson, T. L., Zee, P. C., Loredo, J. S., Ramos, A. R., Singer, R. H., Jimenez, M. C., Barnhart, J. M. & Redline, S. (2015) Periodontitis and Sleep Disordered Breathing in the Hispanic Community Health Study/Study of Latinos. *Sleep* **38,** 1195-1203. doi:10.5665/sleep.4890.

Sanders, A. E., Slade, G. D., Beck, J. D. & Agustsdottir, H. (2011) Secondhand smoke and periodontal disease: atherosclerosis risk in communities study. *Am J Public Health* **101 Suppl 1,** S339-346. doi:10.2105/ajph.2010.300069.

Sanders, A. E., Slade, G. D., Fitzsimmons, T. R. & Bartold, P. M. (2009) Physical activity, inflammatory biomarkers in gingival crevicular fluid and periodontitis. *J Clin Periodontol* **36,** 388-395. doi:10.1111/j.1600-051X.2009.01394.x.

Schmalz, G., Davarpanah, I., Jager, J., Mausberg, R. F., Krohn-Grimberghe, B., Schmidt, J., Haak, R., Sack, U. & Ziebolz, D. (2017) MMP-8 and TIMP-1 are associated to periodontal inflammation in patients with rheumatoid arthritis under methotrexate immunosuppression - First results of a cross-sectional study. *J Microbiol Immunol Infect*. doi:10.1016/j.jmii.2017.07.016.

Schmalz, G., Kollmar, O., Vasko, R., Muller, G. A., Haak, R. & Ziebolz, D. (2016) Oral health-related quality of life in patients on chronic haemodialysis and after kidney transplantation. *Oral Dis* **22,** 665-672. doi:10.1111/odi.12519.

Schurch, E., Minder, C. E., Lang, N. P. & Geering, A. H. (1988) Periodontal Conditions in a Randomly Selected Population in Switzerland. *Community Dentistry and Oral Epidemiology* **16,** 181-186.

Schutzhold, S., Kocher, T., Biffar, R., Hoffmann, T., Schmidt, C. O., Micheelis, W., Jordan, R. & Holtfreter, B. (2015) Changes in prevalence of periodontitis in two German population-based studies. *J Clin Periodontol* **42,** 121-130. doi:10.1111/jcpe.12352.

Shaqman, M., Ioannidou, E., Burleson, J., Hull, D. & Dongari-Bagtzoglou, A. (2010) Periodontitis and inflammatory markers in transplant recipients. *J Periodontol* **81,** 666-672. doi:10.1902/jop.2010.090570.

Shchipkova, A. Y., Nagaraja, H. N. & Kumar, P. S. (2010) Subgingival microbial profiles of smokers with periodontitis. *J Dent Res* **89,** 1247-1253. doi:10.1177/0022034510377203.

Shetty, M., Shetty, P. K., Ramesh, A., Thomas, B., Prabhu, S. & Rao, A. (2010) Periodontal disease in pregnancy is a risk factor for preeclampsia. *Acta Obstet Gynecol Scand* **89,** 718-721. doi:10.3109/00016341003623738.

Shimazaki, Y., Shirota, T., Uchida, K., Yonemoto, K., Kiyohara, Y., Iida, M., Saito, T. & Yamashita, Y. (2008) Intake of dairy products and periodontal disease: the Hisayama Study. *J Periodontol* **79,** 131-137. doi:10.1902/jop.2008.070202.

Skudutyte-Rysstad, R., Slevolden, E. M., Hansen, B. F., Sandvik, L. & Preus, H. R. (2014) Association between moderate to severe psoriasis and periodontitis in a Scandinavian population. *BMC Oral Health* **14,** 139. doi:10.1186/1472-6831-14-139.

Sora, N. D., Marlow, N. M., Bandyopadhyay, D., Leite, R. S., Slate, E. H. & Fernandes, J. K. (2013) Metabolic syndrome and periodontitis in Gullah African Americans with type 2 diabetes mellitus. *J Clin Periodontol* **40,** 599-606. doi:10.1111/jcpe.12104.

Southerland, J. H. (2013) Periodontitis may contribute to poor control of hypertension in older adults. *J Evid Based Dent Pract* **13,** 125-127. doi:10.1016/j.jebdp.2013.07.016.

Susanto, H., Nesse, W., Dijkstra, P. U., Agustina, D., Vissink, A. & Abbas, F. (2011) Periodontitis prevalence and severity in Indonesians with type 2 diabetes. *J Periodontol* **82,** 550-557. doi:10.1902/jop.2010.100285.

Taghzouti, N., Xiong, X., Gornitsky, M., Chandad, F., Voyer, R., Gagnon, G., Leduc, L., Xu, H., Tulandi, T., Wei, B., Senecal, J., Velly, A. M., Salah, M. H. & Fraser, W. D. (2012) Periodontal disease is not associated with preeclampsia in Canadian pregnant women. *J Periodontol* **83,** 871-877. doi:10.1902/jop.2011.110342.

Tervonen, T., Oliver, R. C., Wolff, L. F., Bereuter, J., Anderson, L. A. & Aeppli, D. M. (1994) Prevalence of Periodontal Pathogens with Varying Metabolic Control of Diabetes-Mellitus. *Journal of Clinical Periodontology* **21,** 375-379.

Tsakos, G., Sabbah, W., Hingorani, A. D., Netuveli, G., Donos, N., Watt, R. G. & D'Aiuto, F. (2010) Is periodontal inflammation associated with raised blood pressure? Evidence from a National US survey. *J Hypertens* **28,** 2386-2393. doi:10.1097/HJH.0b013e32833e0fe1.

Usin, M. M., Tabares, S. M., Parodi, R. J. & Sembaj, A. (2013) Periodontal conditions during the pregnancy associated with periodontal pathogens. *J Investig Clin Dent* **4,** 54-59. doi:10.1111/j.2041-1626.2012.00137.x.

Vandervelden, U., Abbas, F., Armand, S., Degraaff, J., Timmerman, M. F., Vanderweijden, G. A., Vanwinkelhoff, A. J. & Winkel, E. G. (1993) The Effect of Sibling Relationship on the Periodontal Condition. *Journal of Clinical Periodontology* **20,** 683-690.

Vettore, M. V., Leal, M., Leao, A. T., da Silva, A. M., Lamarca, G. A. & Sheiham, A. (2008) The relationship between periodontitis and preterm low birthweight. *J Dent Res* **87,** 73-78. doi:10.1177/154405910808700113.

Vlachojannis, C., Dye, B. A., Herrera-Abreu, M., Pikdoken, L., Lerche-Sehm, J., Pretzl, B., Celenti, R. & Papapanou, P. N. (2010) Determinants of serum IgG responses to periodontal bacteria in a nationally representative sample of US adults. *J Clin Periodontol* **37,** 685-696. doi:10.1111/j.1600-051X.2010.01592.x.

Vozza, I., Caldarazzo, V., Polimeni, A. & Ottolenghi, L. (2015) Periodontal disease and cancer patients undergoing chemotherapy. *Int Dent J* **65,** 45-48. doi:10.1111/idj.12133.

Wang, Y. L., Liou, J. D. & Pan, W. L. (2013) Association between maternal periodontal disease and preterm delivery and low birth weight. *Taiwan J Obstet Gynecol* **52,** 71-76. doi:10.1016/j.tjog.2013.01.011.

Wellapuli, N. & Ekanayake, L. (2016) Association between chronic periodontitis and oral health-related quality of life in Sri Lankan adults. *Int Dent J* **66,** 337-343. doi:10.1111/idj.12255.

Wellapuli, N. & Ekanayake, L. (2017) Prevalence, severity and extent of chronic periodontitis among Sri Lankan adults. *Community Dent Health* **34,** 152-156. doi:10.1922/CDH_4070Wellapuli05.

Wu, Y. M., Chuang, H. L., Ho, Y. P., Ho, K. Y. & Tsai, C. C. (2010) Investigation of interleukin-13 gene polymorphisms in individuals with chronic and generalized aggressive periodontitis in a Taiwanese (Chinese) population. *J Periodontal Res* **45,** 695-701. doi:10.1111/j.1600-0765.2010.01287.x.

Xie, C. J., Xiao, L. M., Fan, W. H., Xuan, D. Y. & Zhang, J. C. (2009) Common single nucleotide polymorphisms in cyclooxygenase-2 and risk of severe chronic periodontitis in a Chinese population. *J Clin Periodontol* **36,** 198-203. doi:10.1111/j.1600-051X.2008.01366.x.

Xie, Y., Xiong, X., Elkind-Hirsch, K. E., Pridjian, G., Maney, P., Delarosa, R. L. & Buekens, P. (2014) Prepregnancy obesity and periodontitis among pregnant females with and without gestational diabetes mellitus. *J Periodontol* **85,** 890-898. doi:10.1902/jop.2013.130502.

Ximenez-Fyvie, L. A., Almaguer-Flores, A., Jacobo-Soto, V., Lara-Cordoba, M., Sanchez-Vargas, L. O. & Alcantara-Maruri, E. (2006) Description of the subgingival microbiota of periodontally untreated Mexican subjects: chronic periodontitis and periodontal health. *J Periodontol* **77,** 460-471. doi:10.1902/jop.2006.050177.

Xiong, X., Elkind-Hirsch, K. E., Vastardis, S., Delarosa, R. L., Pridjian, G. & Buekens, P. (2009) Periodontal disease is associated with gestational diabetes mellitus: a case-control study. *J Periodontol* **80,** 1742-1749. doi:10.1902/jop.2009.090250.

Yang, T. H., Masumi, S., Weng, S. P., Chen, H. W., Chuang, H. C. & Chuang, K. J. (2015) Personal exposure to particulate matter and inflammation among patients with periodontal disease. *Sci Total Environ* **502,** 585-589. doi:10.1016/j.scitotenv.2014.09.081.

Yu, Y. H. & Kuo, H. K. (2008) Association between cognitive function and periodontal disease in older adults. *J Am Geriatr Soc* **56,** 1693-1697. doi:10.1111/j.1532-5415.2008.01847.x.

Ziebolz, D., Schmalz, G., Kauffels, A., Widmer, F., Widmer, K., Slotta, J. E., Mausberg, R. F. & Kollmar, O. (2017) Periodontal pathogenic bacteria and aMMP-8 findings depending on periodontal conditions of patients before and after liver transplantation. *Clin Oral Investig* **21,** 745-752. doi:10.1007/s00784-016-1821-4.

*Supplementary Appendix : Radiograph + PD*

Al Yahfoufi, Z. (2017) Prevalence of Periodontal Destruction and Putative Periodontal Pathogens in the Same Lebanese Family. *J Contemp Dent Pract* **18,** 970-976.

Koll-Klais, P., Mandar, R., Leibur, E., Marcotte, H., Hammarstrom, L. & Mikelsaar, M. (2005) Oral lactobacilli in chronic periodontitis and periodontal health: species composition and antimicrobial activity. *Oral Microbiol Immunol* **20,** 354-361. doi:10.1111/j.1399-302X.2005.00239.x.

Lafon, A., Tala, S., Ahossi, V., Perrin, D., Giroud, M. & Bejot, Y. (2014) Association between periodontal disease and non-fatal ischemic stroke: a case-control study. *Acta Odontol Scand* **72,** 687-693. doi:10.3109/00016357.2014.898089.

Modeer, T., Blomberg, C., Wondimu, B., Lindberg, T. Y. & Marcus, C. (2011) Association between obesity and periodontal risk indicators in adolescents. *Int J Pediatr Obes* **6,** e264-270. doi:10.3109/17477166.2010.495779.

Tsakos, G., Bernabe, E., D'Aiuto, F., Pikhart, H., Tonetti, M., Sheiham, A. & Donos, N. (2010) Assessing the minimally important difference in the oral impact on daily performances index in patients treated for periodontitis. *J Clin Periodontol* **37,** 903-909. doi:10.1111/j.1600-051X.2010.01583.x.

*Supplementary Appendix : CAL + furcation*

Prpic, J., Kuis, D., Glazar, I. & Ribaric, S. P. (2013) Association of obesity with periodontitis, tooth loss and oral hygiene in non-smoking adults. *Cent Eur J Public Health* **21,** 196-201.

*Supplementary Appendix : CAL + radiograph*

Al-Habashneh, R., Al-Omari, M. A. & Taani, D. Q. (2009) Smoking and caries experience in subjects with various form of periodontal diseases from a teaching hospital clinic. *Int J Dent Hyg* **7,** 55-61. doi:10.1111/j.1601-5037.2008.00349.x.

Kallestal, C., Matsson, L. & Persson, S. (1991) Proximal attachment loss in Swedish adolescents. *J Clin Periodontol* **18,** 760-765.

Lavu, V., Venkatesan, V., Venkata Kameswara Subrahmanya Lakkakula, B., Venugopal, P., Paul, S. F. & Rao, S. R. (2015) Polymorphic regions in the interleukin-1 gene and susceptibility to chronic periodontitis: a genetic association study. *Genet Test Mol Biomarkers* **19,** 175-181. doi:10.1089/gtmb.2014.0275.

Zhang, Q., Zhang, X., Feng, G., Fu, T., Yin, R., Zhang, L., Feng, X., Li, L. & Gu, Z. (2017) Periodontal disease in Chinese patients with systemic lupus erythematosus. *Rheumatol Int* **37,** 1373-1379. doi:10.1007/s00296-017-3759-5.

*Supplementary Appendix : PD+BOP*

Al Asqah, M., Al Hamoudi, N., Anil, S., Al Jebreen, A. & Al-Hamoudi, W. K. (2009) Is the presence of Helicobacter pylori in dental plaque of patients with chronic periodontitis a risk factor for gastric infection? *Can J Gastroenterol* **23,** 177-179.

al-Yahfoufi, Z., Mombelli, A., Wicki, A. & Lang, N. P. (1995) The effect of plaque control in subjects with shallow pockets and high prevalence of periodontal pathogens. *J Clin Periodontol* **22,** 78-84.

Chambrone, L., Ascarza, A., Guerrero, M. E., Pannuti, C., de la Rosa, M., Salinas-Prieto, E. & Mendoza, G. (2014) Association of -1082 interleukin-10 gene polymorphism in Peruvian adults with chronic periodontitis. *Med Oral Patol Oral Cir Bucal* **19,** e569-573.

Gluhak, C., Arnetzl, G. V., Kirmeier, R., Jakse, N. & Arnetzl, G. (2010) Oral status among seniors in nine nursing homes in Styria, Austria. *Gerodontology* **27,** 47-52. doi:10.1111/j.1741-2358.2009.00281.x.

Kubota, M., Tanno-Nakanishi, M., Yamada, S., Okuda, K. & Ishihara, K. (2011) Effect of smoking on subgingival microflora of patients with periodontitis in Japan. *BMC Oral Health* **11,** 1. doi:10.1186/1472-6831-11-1.

Mineoka, T., Awano, S., Rikimaru, T., Kurata, H., Yoshida, A., Ansai, T. & Takehara, T. (2008) Site-specific development of periodontal disease is associated with increased levels of Porphyromonas gingivalis, Treponema denticola, and Tannerella forsythia in subgingival plaque. *J Periodontol* **79,** 670-676. doi:10.1902/jop.2008.070398.

Nuernberg, M. A. A., Rodrigues, S. C., Perdoncini, N. N., Funke, V. A. M., Bonfim, C. M. S., Nabhan, S. K. & Torres-Pereira, C. C. (2017) Periodontal status of candidates for allogeneic hematopoietic stem cell transplantation. *Spec Care Dentist* **37,** 187-193. doi:10.1111/scd.12229.

*Supplementary Appendix : PD+ Furcation + Radiograph*

Folwaczny, M., Glas, J., Torok, H. P., Tonenchi, L., Paschos, E., Malachova, O., Bauer, B. & Folwaczny, C. (2005) Prevalence of the -295 T-to-C promoter polymorphism of the interleukin (IL)-16 gene in periodontitis. *Clin Exp Immunol* **142,** 188-192. doi:10.1111/j.1365-2249.2005.02902.x.

*Supplementary Appendix : PD+ CAL+ Radiograph*

Al Habashneh, R., Alchalabi, H., Khader, Y. S., Hazza'a, A. M., Odat, Z. & Johnson, G. K. (2010) Association between periodontal disease and osteoporosis in postmenopausal women in jordan. *J Periodontol* **81,** 1613-1621. doi:10.1902/jop.2010.100190.

Nibali, L., Atkinson, C., Griffiths, P., Darbar, U., Rakmanee, T., Suvan, J. & Donos, N. (2009) Low prevalence of subgingival viruses in periodontitis patients. *J Clin Periodontol* **36,** 928-932. doi:10.1111/j.1600-051X.2009.01476.x.

Reddy, M. S., Geurs, N. C., Jeffcoat, R. L., Proskin, H. & Jeffcoat, M. K. (2000) Periodontal disease progression. *J Periodontol* **71,** 1583-1590. doi:10.1902/jop.2000.71.10.1583.

*Supplementary Appendix : PD+CAL+BOP*

Cruz, S. S., Costa Mda, C., Gomes-Filho, I. S., Barreto, M. L., dos Santos, C. A., Martins, A. G., Passos Jde, S., de Freitas, C. O., Sampaio, F. P. & Cerqueira Ede, M. (2010) Periodontal therapy for pregnant women and cases of low birthweight: an intervention study. *Pediatr Int* **52,** 57-64. doi:10.1111/j.1442-200X.2009.02888.x.

da Silva-Boghossian, C. M., do Souto, R. M., Luiz, R. R. & Colombo, A. P. (2011) Association of red complex, A. actinomycetemcomitans and non-oral bacteria with periodontal diseases. *Arch Oral Biol* **56,** 899-906. doi:10.1016/j.archoralbio.2011.02.009.

de Carvalho, H. L., Thomaz, E. B., Alves, C. M. & Souza, S. F. (2016) Are sickle cell anaemia and sickle cell trait predictive factors for periodontal disease? A cohort study. *J Periodontal Res* **51,** 622-629. doi:10.1111/jre.12342.

Diniz Barreto, L. P., Melo Dos Santos, M., Gomes Bda, S., Lamas Cda, C., Silva, D. G., Silva-Boghossian, C. M., Soares, L. G. & Vieira Falabella, M. E. (2016) Periodontal Conditions in Human Immunodeficiency Virus-Positive Patients Under Highly Active Antiretroviral Therapy From a Metropolitan Area of Rio De Janeiro. *J Periodontol* **87,** 338-345. doi:10.1902/jop.2015.150345.

Esteves Lima, R. P., Miranda Cota, L. O. & Costa, F. O. (2013) Association between periodontitis and gestational diabetes mellitus: a case-control study. *J Periodontol* **84,** 1257-1265. doi:10.1902/jop.2012.120350.

Figueredo, C. M., Brito, F., Barros, F. C., Menegat, J. S., Pedreira, R. R., Fischer, R. G. & Gustafsson, A. (2011) Expression of cytokines in the gingival crevicular fluid and serum from patients with inflammatory bowel disease and untreated chronic periodontitis. *J Periodontal Res* **46,** 141-146. doi:10.1111/j.1600-0765.2010.01303.x.

Gomes-Filho, I. S., das Merces, M. C., de Santana Passos-Soares, J., Seixas da Cruz, S., Teixeira Ladeia, A. M., Trindade, S. C., de Moraes Marcilio Cerqueira, E., Freitas Coelho, J. M., Marques Monteiro, F. M., Barreto, M. L., Pereira Vianna, M. I., Nascimento Costa Mda, C., Seymour, G. J. & Scannapieco, F. A. (2016a) Severity of Periodontitis and Metabolic Syndrome: Is There an Association? *J Periodontol* **87,** 357-366. doi:10.1902/jop.2015.150367.

Gomes-Filho, I. S., Pereira, E. C., Cruz, S. S., Adan, L. F., Vianna, M. I., Passos-Soares, J. S., Trindade, S. C., Oliveira, E. P., Oliveira, M. T., Cerqueira Ede, M., Pereira, A. L., Barreto, M. L. & Seymour, G. J. (2016b) Relationship Among Mothers' Glycemic Level, Periodontitis, and Birth Weight. *J Periodontol* **87,** 238-247. doi:10.1902/jop.2015.150423.

Gomes-Filho, I. S., Santos, C. M., Cruz, S. S., Passos Jde, S., Cerqueira Ede, M., Costa Mda, C., Santana, T. C., Seymour, G. J., Santos, C. A. & Barreto, M. L. (2009) Periodontitis and nosocomial lower respiratory tract infection: preliminary findings. *J Clin Periodontol* **36,** 380-387. doi:10.1111/j.1600-051X.2009.01387.x.

Gomes-Filho, I. S., Soledade-Marques, K. R., Seixas da Cruz, S., de Santana Passos-Soares, J., Trindade, S. C., Souza-Machado, A., Fischer Rubira-Bullen, I. R., de Moraes Marcilio Cerqueira, E., Barreto, M. L., Costa de Santana, T. & Freitas Coelho, J. M. (2014) Does periodontal infection have an effect on severe asthma in adults? *J Periodontol* **85,** e179-187. doi:10.1902/jop.2013.130509.

Goncalves, C., Soares, G. M., Faveri, M., Perez-Chaparro, P. J., Lobao, E., Figueiredo, L. C., Baccelli, G. T. & Feres, M. (2016) Association of three putative periodontal pathogens with chronic periodontitis in Brazilian subjects. *J Appl Oral Sci* **24,** 181-185. doi:10.1590/1678-775720150445.

Goncalves, L. S., Ferreira, S. M., Souza, C. O. & Colombo, A. P. (2009) Influence of IL-1 gene polymorphism on the periodontal microbiota of HIV-infected Brazilian individuals. *Braz Oral Res* **23,** 452-459.

Grenier, G., Gagnon, G. & Grenier, D. (2009) Detection of herpetic viruses in gingival crevicular fluid of patients suffering from periodontal diseases: prevalence and effect of treatment. *Oral Microbiol Immunol* **24,** 506-509. doi:10.1111/j.1399-302X.2009.00542.x.

Ishaan, D., Parthasarathy, H., Ponnaiyan, D. & Tadepalli, A. (2017) The CD14 rs2569190 TT Genotype is Associated with Chronic Periodontitis. *Genet Test Mol Biomarkers* **21,** 560-564. doi:10.1089/gtmb.2017.0029.

Kissa, J., Chemlali, S., El Houari, B., Amine, K., Khlil, N., Mikou, S., Nadifi, S. & Albandar, J. M. (2016) Aggressive and chronic periodontitis in a population of Moroccan school students. *J Clin Periodontol* **43,** 934-939. doi:10.1111/jcpe.12584.

Michalowicz, B. S., Hodges, J. S., DiAngelis, A. J., Lupo, V. R., Novak, M. J., Ferguson, J. E., Buchanan, W., Bofill, J., Papapanou, P. N., Mitchell, D. A., Matseoane, S. & Tschida, P. A. (2006) Treatment of periodontal disease and the risk of preterm birth. *N Engl J Med* **355,** 1885-1894. doi:10.1056/NEJMoa062249.

Novak, K. F., Taylor, G. W., Dawson, D. R., Ferguson, J. E., 2nd & Novak, M. J. (2006) Periodontitis and gestational diabetes mellitus: exploring the link in NHANES III. *J Public Health Dent* **66,** 163-168.

Schwarzberg, K., Le, R., Bharti, B., Lindsay, S., Casaburi, G., Salvatore, F., Saber, M. H., Alonaizan, F., Slots, J., Gottlieb, R. A., Caporaso, J. G. & Kelley, S. T. (2014) The personal human oral microbiome obscures the effects of treatment on periodontal disease. *PLoS One* **9,** e86708. doi:10.1371/journal.pone.0086708.

Silva-Boghossian, C. M., Orrico, S. R., Goncalves, D., Correa, F. O. & Colombo, A. P. (2014) Microbiological changes after periodontal therapy in diabetic patients with inadequate metabolic control. *Braz Oral Res* **28**.

Soledade-Marques, K. R., Gomes-Filho, I. S., da Cruz, S. S., Passos-Soares, J. S., Trindade, S. C., Cerqueira, E. M. M., Coelho, J. M. F., Barreto, M. L., Costa, M., Vianna, M. I. P., Scannapieco, F. A., Cruz, A. A. & Souza-Machado, A. (2017) Association between periodontitis and severe asthma in adults: A case-control study. *Oral Dis*. doi:10.1111/odi.12737.

Souto, R. & Colombo, A. P. (2008) Prevalence of Enterococcus faecalis in subgingival biofilm and saliva of subjects with chronic periodontal infection. *Arch Oral Biol* **53,** 155-160. doi:10.1016/j.archoralbio.2007.08.004.

*Supplementary Appendix : PD+ Radiograph +BOP*

Bengtsson, V. W., Persson, G. R., Berglund, J. & Renvert, S. (2016) A cross-sectional study of the associations between periodontitis and carotid arterial calcifications in an elderly population. *Acta Odontol Scand* **74,** 115-120. doi:10.3109/00016357.2015.1050603.

Camelo-Castillo, A. J., Mira, A., Pico, A., Nibali, L., Henderson, B., Donos, N. & Tomas, I. (2015) Subgingival microbiota in health compared to periodontitis and the influence of smoking. *Front Microbiol* **6,** 119. doi:10.3389/fmicb.2015.00119.

*Supplementary Appendix : Edema +BOP +PD+ recession +mobility*

Bonner, M., Amard, V., Bar-Pinatel, C., Charpentier, F., Chatard, J. M., Desmuyck, Y., Ihler, S., Rochet, J. P., Roux de La Tribouille, V., Saladin, L., Verdy, M., Girones, N., Fresno, M. & Santi-Rocca, J. (2014) Detection of the amoeba Entamoeba gingivalis in periodontal pockets. *Parasite* **21,** 30. doi:10.1051/parasite/2014029.
